# Clinical Implications of Necroptosis Genes Expression for Cancer Immunity and Prognosis: A Pan-Cancer Analysis

**DOI:** 10.3389/fimmu.2022.882216

**Published:** 2022-06-20

**Authors:** Xin-yu Li, Li-xin Su, Wen-Xue Chen, Hui Liu, Lu-yu Zhang, Yu-Chen Shen, Jian-Xiong You, Jing-Bing Wang, Liming Zhang, Deming Wang, Ming-Zhe Wen, Zhenfeng Wang, Yu-hao Shao, De-Hu Chen, Xi-tao Yang

**Affiliations:** ^1^ Department of Interventional Therapy, Shanghai Ninth People’s Hospital, Shanghai Jiao Tong University School of Medicine, Shanghai, China; ^2^ Department of Neurosurgery, Shanghai Ninth People’s Hospital, Shanghai JiaoTong University School of Medicine, Shanghai, China; ^3^ Department of Nephrology, Shanghai Jiao Tong University Affiliated Sixth People’s Hospital, Shanghai, China; ^4^ The Department of Kidney Transplantation, The First Affiliated Hospital of Zhengzhou University, Zhengzhou, China; ^5^ Department of Ophthalmology, Shanghai Tenth People’s Hospital, Tongji University, Shanghai, China; ^6^ Department of Gastrointestinal Surgery, Hospital Affiliated 5 to Nantong University (Taizhou People's Hospital), Taizhou, China

**Keywords:** cancer, necroptosis fate decisions, pan-cancer, genes, tumor

## Abstract

**Background:**

Necroptosis, a form of programmed cell death, is increasingly being investigated for its controversial role in tumorigenesis and progression. Necroptosis suppresses tumor formation and tumor development by killing tumor cells; however, the necrotic cells also promote tumor formation and tumor development *via* the immunosuppressive effect of necroptosis and inflammatory response caused by cytokine release. Thus, the exact mechanism of necroptosis in pan-cancer remains unknown.

**Methods:**

The data of 11,057 cancer samples were downloaded from the TCGA database, along with clinical information, tumor mutation burden, and microsatellite instability information of the corresponding patients. We used the TCGA data in a pan-cancer analysis to identify differences in mRNA level as well as single nucleotide variants, copy number variants, methylation profiles, and genomic signatures of miRNA-mRNA interactions. Two drug datasets (from GDSC, CTRP) were used to evaluate drug sensitivity and resistance against necroptosis genes.

**Results:**

Necroptosis genes were aberrantly expressed in various cancers. The frequency of necroptosis gene mutations was highest in lung squamous cell carcinoma. Furthermore, the correlation between necroptosis gene expression in the tumor microenvironment and immune cell infiltration varied for different cancers. High necroptosis gene expression was found to correlate with NK, Tfh, Th1, CD8_T, and DC cells. These can therefore be used as biomarkers to predict prognosis. By matching gene targets with drugs, we identified potential candidate drugs.

**Conclusion:**

Our study showed the genomic alterations and clinical features of necroptosis genes in 33 cancers. This may help clarify the link between necroptosis and tumorigenesis. Our findings may also provide new approaches for the clinical treatment of cancer.

## Introduction

In multicellular organisms, the balance between cell proliferation and cell death is essential to maintain physiological homeostasis and is one of the most important conditions for growth and development. The incidence of malignant tumors is significantly increased when either excessive cell proliferation or normal cell death is inhibited. Therefore, some researchers posit that the two distinguishing features of malignant tumors are unrestricted cell proliferation and inhibition of death (1). Necroptosis is a programmed cell death that is distinct from the classical apoptotic pathway dependent on caspase activation. When caspases are absent or inhibited, the classical apoptosis is inhibited, with necroptosis activated as the alternative. Existing studies suggest that necroptosis both inhibits and promotes the development of cancer. Previous studies found that necroptosis was associated with poor survival in ovarian, colorectal, and breast cancers (2–4). On the other hand, necroptosis can also contribute to tumor development and metastasis through a variety of mechanisms. For example, since necroptosis is pro-inflammatory cell death, the inflammatory response may lead to metastasis in breast cancer (5). However, the genomic necroptosis in cancer require further study.

In this study, we analyzed the differences in expression of necroptosis genes in pan-cancerous tissues using several databases such as The Cancer Genome Atlas (TCGA) and preliminarily investigated their predictive value for tumor prognosis as well as their involvement in regulating multiple tumor-related immune responses. We also investigated the epigenetic regulation of necroptosis genes to relate potential altered methylation status to prognostic outcomes. Our study provides a theoretical basis for further clarification of potential tumor immunotherapy targets.

## Materials and Methods

### Data Download and Processing

**Figure 1:**
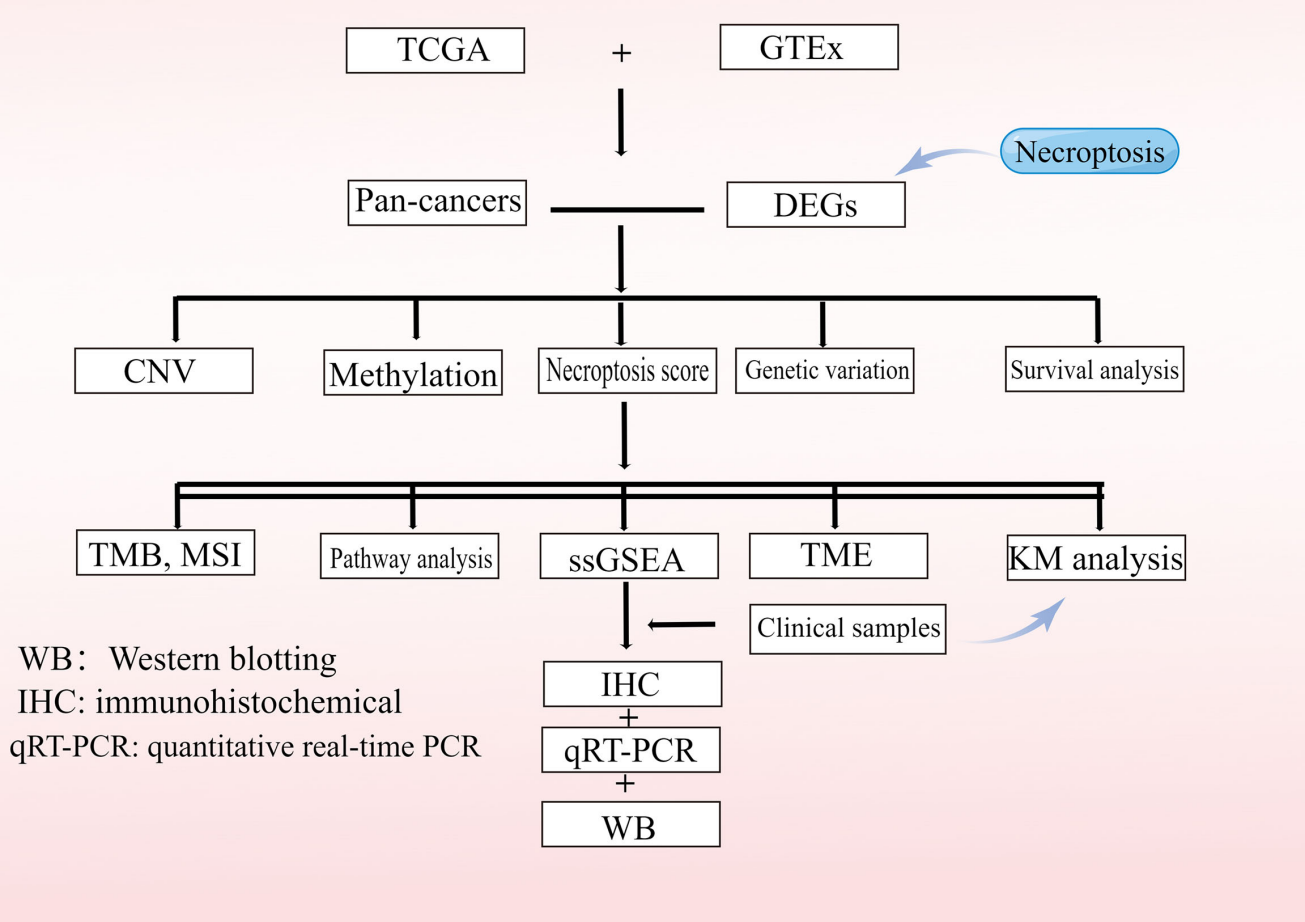
Flowchart of the study.

Data from 11,057 samples including 33 tumor types were downloaded from the UCSC Xena GDC TCGA database(Date of data update: 2021.5.17), including expression profiles, survival data, copy number variation (CNV) data (*n*=11,495), single-nucleotide variation (SNV) data (*n*=8,663), and methylation data (*n*=10,129) for these genes (6). In addition, demographic information, tumor information, and follow-up data were obtained for all patients. A total of 52 necroptosis genes were identified from were obtained from the GSEA website (https://www.gsea-msigdb.org/gsea) and prior reviews (7–9). In this study, 33 TCGA tumor types and abbreviations were used for international common terms. To analyze normal tissue expressions, we used the Genotype-Tissue Expression Portal (GTEx; https://www.gtexportal.org/home/) (10) and gene expression values were quantitated as transcripts per million (TPM). Variance analysis using the R language “limma” package, extracting *P*-values and log|fold-change (FC)| of genes for heat map presentation. The study flowchart is presented in **Figure 1**. The list of abbreviations were presented in **Supplementary Table 1**.

### Survival Analysis

Necroptosis gene expression data from 33 tumors were combined with their corresponding clinical survival data for expression-based survival analysis. Tumor specimens were divided into two groups (high and low) based on the median of the “RSEM” package. The R “survival” package was used to adjust the survival time and survival status of the two groups. The survival risk (hazard ratio, HR) was calculated for each gene using a Cox proportional hazard model. A log-rank test for Kaplan-Meier survival was performed for each gene. Genes with a *P*-value less than 0.05 for the log-rank test were retained.

### Single-Nucleotide Variation Analysis

SNV data were collected from the TCGA database for 33 cancers (*n*=8,663). The downloaded data included the following variant types: Missense_Mutation, Silent, 5’ Flank, 3’ UTR, RNA, In_Frame_Del, Nonsense_Mutation, Splice_Site, Intron, 5’ UTR, In_Frame_Ins, Frame_Shift_Del, Nonstop_Mutation, 3’ Flank, Frame_Shift_Ins, and Translation_Start_Site. The number of mutated samples/number of tumor samples were used to calculate the frequency (percentage) of SNV mutations in the coding region of each gene and an SNV oncoplot was generated using the R package “maftools” (11).

### Copy Number Variation Analysis

CNVs are the major aberrations that lead to changes in gene expression during tumorigenesis and tumor growth (12). CNVs are divided into two subtypes, homozygous and heterozygous, which include amplifications and deletions. Heterozygous variants indicate that CNVs occurred on one chromosome, while homozygous variants define variants on both chromosomes. Raw data for 33 cancers with CNVs (*n*=11495) were collected from the TCGA database using GISTIC v2.0 to extract the required fragment data, synthesize the signaling data, and combine the data with human reference genome data to obtain gene CNVs. To determine the percentage of amplification and deletion of CNVs for necroptosis genes in pan-cancer, percentage statistics were generated based on CNV isoforms with GISTIC-processed CNV data, and correlations with raw CNV data as well as mRNA-RSEM data were calculated—considering only genes with CNVs above 5% as significant variants (11, 13). Associations between paired mRNA levels and the percentage of paired CNV samples were calculated based on Pearson’s product-moment correlation coefficient and *t*-distribution *(* 14). *P*-values ​​were false discovery rate (FDR)-adjusted.

### Methylation Analysis

The abnormal expression of tumor genes is mainly due to the abnormal methylation of regulatory regions (15).To examine methylation data for tumor and normal tissue pairs, we collected methylation data from the TCGA database (*n*=10,129). Only 14 cancer types had paired data; hence, the differential methylation analysis is based on these 14 cancer types. To determine the significant difference in methylation between tumor and normal samples, the Student’s *t*-test was performed and an FDR-adjusted *P ≤* 0.05 was considered significant. Following the method of a previous publication, we combined overall survival (OS) and methylation data (11). Gene methylation levels were divided into two groups based on average methylation levels. If the Cox coefficient was >0, the hypermethylated group had poor survival defined as high risk, otherwise, it was defined as low risk. The distribution of the two groups was tested logarithmically to obtain the sum of ranks at *P*<0.05.

### Pathway Activity Analysis

Reverse-phase protein array (RPPA) data from the TCPA database was used to calculate scores for 7,876 samples, 10 cancer-related pathways, and 32 cancer types. RPPA is a high-throughput antibody-based assay with similar procedures to western blotting. Proteins were extracted from tumor tissue or cultured cells, denatured with SDS, plated on nitrocellulose-coated slides, and subsequently probed with antibodies (TCPA database). All TCPA RPPA data were obtained from the TCGA samples. Pathways of interest included the TSC/mTOR, RTK, RAS/MAPK, PI3K/AKT, estrogen receptor (ER) hormone, androgen receptor (AR) hormone, epithelial-mesenchymal transition (EMT), DNA damage response, cell cycle, and apoptosis pathways. These are all known pathways associated with cancer (16). Replicates-based normalized RPPA data were median-centered and standard deviation normalized across all samples for each component to give relative protein levels. Gene expression data were divided into two groups (groupHigh and groupLow) by median expression. The significant difference in pathway activity score (PAS) between the groups was determined by a *t*-test, with FDR-corrected *P* ≤ 0.05 considered significant. For instance, if PAS (Gene A groupHigh) >PAS (Gene A groupLow), we considered that Gene A may have an activating effect on the pathway, otherwise it would have an inhibitory effect (17).

### MicroRNA (miRNA) Regulatory Network Analysis

Expression data for miRNA transcripts were collected from the TCGA database, including that of 9,105 samples and 33 cancer types. Experimentally validated data (from scientific papers, TarBase, miRTarBase, and miR2Disease), as well as data from Target Scan and Miranda predictions, were included. Only miRNA pairs of genes that have been recorded were used to calculate the expression correlation. Subsequently, gene expression and miRNA levels were then correlated with barcodes from TCGA probes. Pearson correlation coefficients and *t*-distributions were used to calculate correlations between paired mRNA and miRNA levels. After determining the FDR-corrected *P*-values (FDR-cutoff <0.05 and *R*<0), correlations were calculated for all paired samples, and transcription factors were identified as positive regulators, whereas negatively correlated miRNA-gene pairs were considered as potential negative regulators. Finally, rule maps were constructed using the “VisNetwork” R package.

### Drug Sensitivity Analysis

We collected the half-maximal inhibitory concentration (IC_50_) values of 265 small molecules from the Genomics of Drug Sensitivity in Cancer (GDSC) database in 860 cell lines along with their corresponding mRNA levels, and that of 481 small molecules from the Cancer Therapeutics Response Portal (CTRP) database in 1,001 cell lines, along with their corresponding mRNA levels. The mRNA and drug sensitivity data were combined. Pearson correlation analysis was used to determine the correlation between mRNA levels and drug IC_50_ values. *P*-values were adjusted using FDR<0.05. Bubble plots were used to summarize the correlation between genes and drugs.

## Results

### Gene Expression and Typing Analysis of Necroptosis Genes

**Figure 2:**
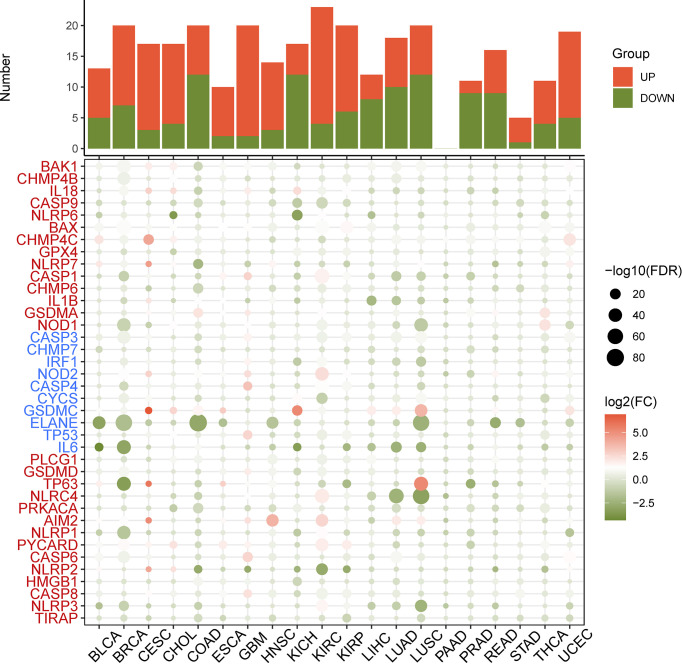
Heatmap of the necroptosis gene expression profiles in the TCGA dataset. Red: increased expression, green: decreased expression and the circles size represent the false-discovery rate (FDR).

**Figure 3:**
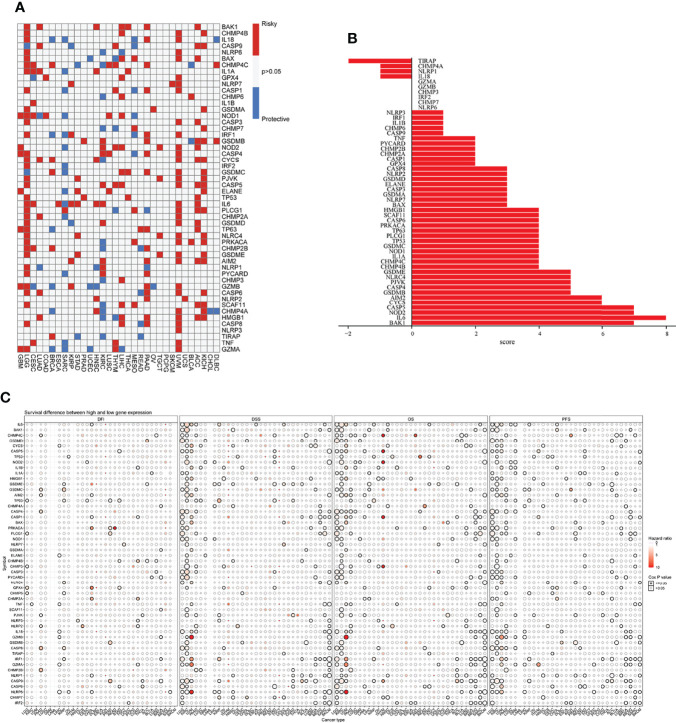
**(A)** Univariate Cox regression analysis of necroptosis genes in pan-cancer. Grey denotes *P*>0.05, red represents *P*<0.05 (HR>1), while blue denotes HR<1. **(B)** The number of genes with significant Cox analysis in all tumors was counted. The final risk score was determined by adding risk factors (+1) and subtracting protective factors (–1). **(C)** Survival difference between high and low gene expression groups. The size of the dots represents the significance of the gene’sinfluence on survival for each cancer type, and the statistical significance of differences was determined by cox regression analysis. Red dots represent worse survival and blue dots represent the opposite.

First, the differential expression of necroptosis genes was compared between all normal tissues according to the GTEx database. We found that the necroptosis genes were widely expressed in a variety of tissues, with *HMGB1, GPX4, CHMP4B*, and *CHMP2A* being highly expressed in normal tissues *in vivo*, and *GPX4* being most highly expressed in the testis (**Supplementary Figure 1**). Subsequently, using the TCGA expression data, we tested the differential expression of necroptosis genes in a pan-cancer analysis. Differential expression analysis revealed aberrantly expressed genes in 20 solid tumors, with individual genes differentially expressed in different tumors (**Figure 2**). For example, *TP63* was lowly expressed in breast cancer (BRCA) and highly expressed in lung squamous cell carcinoma (LUSC). Gene expression also differed in the same tumor, with *IL6* and *NLRP6* being low expressed in kidney chromophobe (KICH) while *GSDMC* was high. Differential expression of different necroptosis genes may imply differences in the mechanisms by which they control tumor development. We next evaluated the relationship between gene expression and immune cell infiltration. Next, we performed a one-way Cox analysis for each gene. **Figure 3A** shows the results of one-way Cox analysis (*P*>0.05 in gray; *P*<0.05, HR>1 in red; HR<1 in blue). The number of genes with significant Cox analysis in all tumors was counted. The final risk score was determined by adding risk factors (+1) and subtracting protective factors (–1) (**Figure 3B**). We found that most of the necroptosis genes had risk scores greater than 0, suggesting an important role in tumor development. We combined groups of patients with high and low necroptosis gene expression with patient survival outcomes to determine their association. Survival rates were classified into overall survival (OS), progression-free survival (PFS), disease-specific survival (DSS), and disease-free survival (DFS). The results of the analysis showed that the genes were closely associated with the prognostic indicators of patients, and most of them were risk factors (heatmap shown in **Figure 3C**). For example, *PLCG1* in low-grade gliomas (LGGs) is associated with worse prognostic indicators (for OS, DSS, PFS, DFS) in patients. To make the results more convincing, univariable and multivariable Cox regression analyses were performed to assess independent prognostic factors for survival. In LGG, we found that *CASP9*, *PLCG1*, and *TP53* remained statistically significant in multivariate Cox analysis. suggesting that these genes are independent prognostic factors. Also, our results suggest that *CASP9* is an independent risk factor in ACC after the inclusion of other clinical indicators (**Supplementary Table 2**). These results suggest that dysregulation of necroptosis gene expression may be involved in tumorigenesis. Gene expression subtype analysis showed that different genes affect different cancer subtypes. To identify clinically relevant genes affecting cancer subtypes, we analyzed the expression of each gene subtype in different cancer subtypes. We found significant differences in most necroptosis genes in the subtypes of stomach adenocarcinoma (STAD), kidney renal clear cell carcinoma (KIRC), LUSC, lung adenocarcinoma (LUAD), BRCA, as well as head and neck cancer (HNSC) but no differences in bladder urothelial carcinoma (BLCA) subtypes (**Supplementary Figure 2**).

### Genetic Correlation Analysis and Genetic Variation

We first analyzed the correlations between necroptosis genes and found positive correlations between most necroptosis genes, suggesting a functional synergy between them (**Supplementary Figure 3A**). Notably, thyroid cancer (THCA) samples exhibited the highest number of variant genes (*n*=404, **Supplementary Figure 3B**). Gene amplification was predominant in BRCA, whereas mutations were predominant in STAD, colon adenocarcinoma (COAD), and rectum adenocarcinoma (READ) (**Supplementary Figure 3B**). Although the cause of tumors is currently unknown, mutations and amplification of both oncogenes and tumor suppressor genes are associated with tumor development (18).

**Figure 4:**
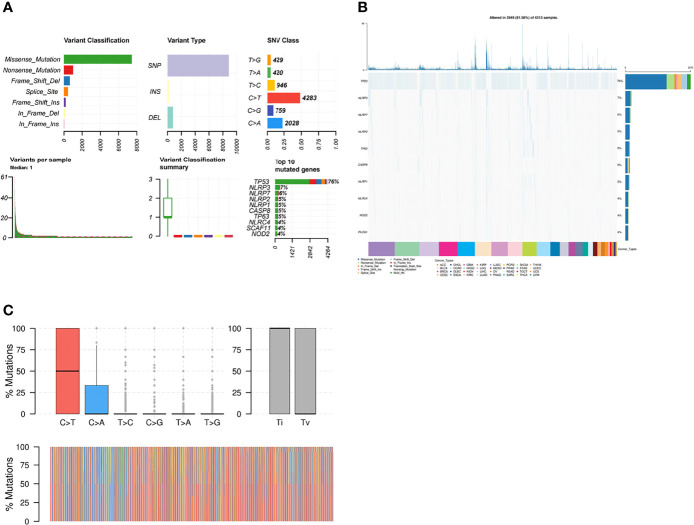
**(A)** Figure summarizing the SNV classes of necroptosis gene set in pan-cancers. **(B)** Oncoplot of SNVs of top 10 mutated genes from the gene set of specific cancers. **(C)** Transition (Ti) and transversion (Tv) classification of the SNVs of necroptosis genes in pan cancers.

Then, we analyzed the mutation frequency of the different necroptosis genes using a pan-cancer analysis. The frequency of SNVs of individual genes associated with necroptosis genes ranged from 1–86% in different cancer types, of which *TP53* had the highest percentage (**Supplementary Figure 4**). The most common variant classification was Missense_Mutation and the most common variant type was SNPs (**Figure 4A**). Furthermore, single SNVs were dominated by C>T (**Figure 4A**). Cascade plots were used to analyze specific mutation types. As shown in **Figure 4B**, the total SNV frequency of the genes was 91.56% (3,949/4,313 samples). their corresponding mutation percentages were *TP53* (76%)*, NLRP3* (7%)*, NLRP7* (6%)*, NLRP2* (5%)*, TP63* (5%)*, CASP8* (5%)*, NLRP1* (5%)*, NLRC4* (4%)*, NOD2* (4%), and *PLCG1* (4%) (**Figure 4B**). SNV frequency of the necroptosis genes was increased in sarcoma (SARC), LUSC, LUAD, and BRCA. Moreover, the mutations were classified into transitions (Ti) and transversions (Tv). **Figure 4C** shows the distribution of Ti and Tv of necroptosis gene sets in the sample set of pan-cancer types for SNV classifications based on C>T and C>A. SNV survival analysis revealed significant differences in survival between mutated and non-mutated genes, e.g., mutated and non-mutated genes in *BAX* in BRCA, and *NLRP7* in mesothelioma (MESO) (**Supplementary Figure 5**).

### Copy Number Variation

**Figure 5:**
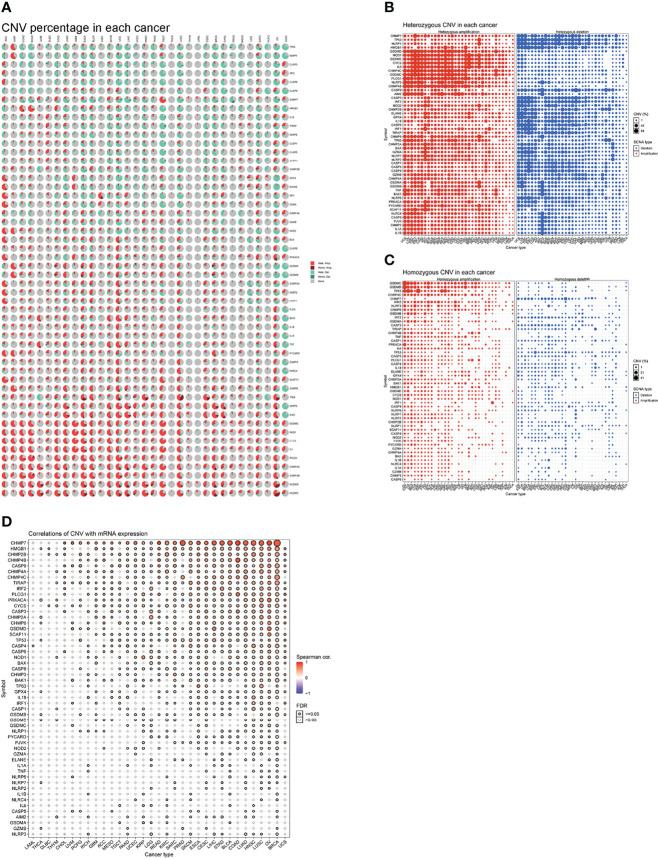
**(A)** A pie chart representing the proportion of different types of CNV of one gene in one cancer, and different colors represent different types of CNV. Hete Amp = heterozygous amplification; Hete Del = heterozygous deletion; Homo Amp = homozygous amplification; Homo Del = homozygous deletion; None = no CNV. **(B)** Heterozygous CNV profile, including amplification and deletion percentage for each gene in each cancer. Only genes with >5% CNV in cancers are shown. The size of the point represents the statistical significance, where the bigger the dot size, the higher the statistical significance. FDR, false discovery rate. **(C)** Homozygous CNV profile, including amplification and deletion percentage for each gene in each cancer. Only genes with >5% CNV in cancers are shown. The size of the point represents the statistical significance, where the bigger the dot size, the higher the statistical significance. FDR, false discovery rate. **(D)** mRNA expression and CNV data merged by TCGA barcode. The association between paired mRNA expression and CNV percent samples was tested based on Pearson’s product-moment correlation coefficient and follows a *t*-distribution. The size of the point represents the statistical significance, where the bigger the dot size, the higher the statistical significance. FDR, false discovery rate.

To detect CNV changes in genes involved in necroptosis at the chromosomal level, we analyzed CNV data for necroptosis genes in the TCGA database. **Figure 5A** shows a pie chart of 33 cancer types indicating the main CNV of the necroptosis genes was heterozygous amplification or deletion. CNV percentage analysis showed heterozygous amplification of *TP53* in kidney renal papillary cell carcinoma (KIRP); as well as *GSDMD*, *NOD1*, *GSDME*, *CYCS*, and *IL6* in Tenosynovial giant cell tumor (TGCT), uterine carcinosarcomas (UCS), ovarian cancer (OV), LUSC, LUAD, esophageal carcinoma (ESCA), and adenoid cystic carcinoma (ACC) of greater than 84%. Furthermore, heterozygous deletion of *CHMP7* in LUSC, UCS, and OV; *GPX4* in UCS, OV; as well as *NLRC*4 in liver hepatocellular carcinoma (LICH) was all greater than 84% (**Figure 5B**). In addition, homozygous amplification and deletion were analyzed. *GSDMC* and *GSDMD* in OV and *IRF1* in KIRC subtypes had greater than 84% homozygous amplification, while *CHMP7* in prostate adenocarcinoma (PRAD) had greater than 84% homozygous deletion levels (**Figure 5C**). Correlation analysis showed that mRNA levels were positively correlated with CNV, in particular, *CHMP7* in BRCA and *CHMP4B* in COAD; however, *CASP5* in uveal melanoma (UVM) and *AIM2* in cholangiocarcinoma (CHOL) were negatively correlated. These results suggest that the CNV of genes mediates their aberrant expression. This may play an important role in cancer progression (**Figure 5D**).

### Methylation Analysis

**Figure 6:**
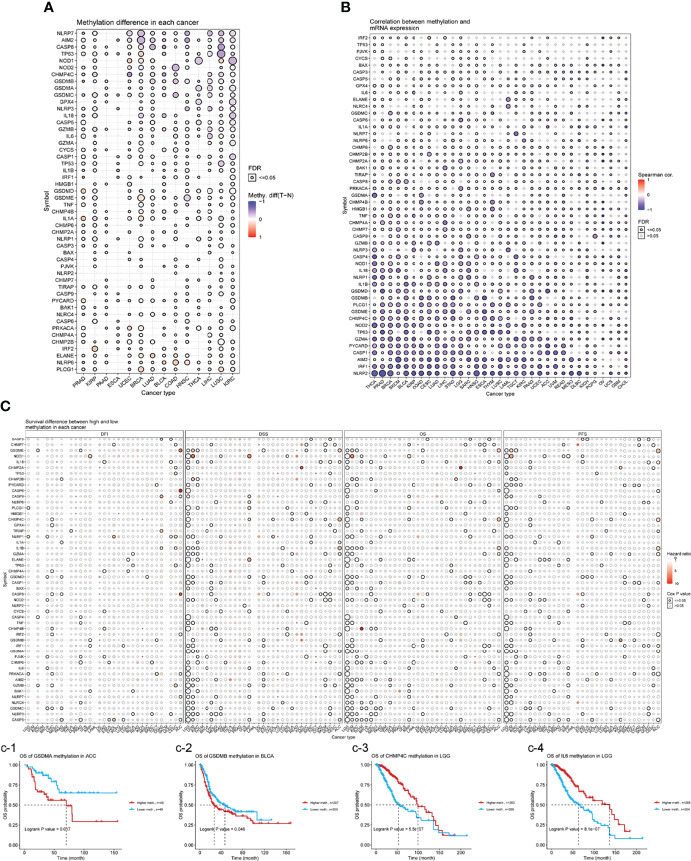
**(A)** Differential methylation in necroptosis-related genes between tumor (T) and normal (N) samples in each cancer. Red dots represent increased methylation in tumors and blue dots represent decreased methylation in tumors. The darker the dot color, the larger the difference in methylation level. **(B)** Correlations between methylation and mRNA levels of genes in specific cancers. Red points represent a positive correlation, and blue dots represent a negative orrelation. The size of the point represents the statistical significance, where the bigger the dot size, the higher the statistical significance. FDR, false discovery rate. **(C)** Survival difference between necroptosis-related genes with high and low methylation levels and samples. Red dots represent worse survival of the hypermethylation group; blue dots represent the opposite. The dot size represents the statistical significance, the larger the dot size means, the higher the statistical significance.

Epigenetic modifications play an important role in tumorigenesis and cancer progression (19). Hence, we explored the methylation of necroptosis genes to identify epigenetic regulation. As shown in **Figure 6A**, necroptosis gene methylation in different tumors is very heterogeneous. In KIRC and LUSC cancers, there were more hypomethylated genes than hypermethylated genes; while in PRAD, KIRP, and BRCA subtypes, there were more hypermethylated genes than hypomethylated genes. *CHMP4C* and *CASP5* were hypomethylated in most cancer types, while *PLCG1* and *NLRP6* were hypermethylated in most cancer types (**Figure 6A**). Correlation analysis of methylation and mRNA level showed that the expression of most genes was negatively correlated with their methylation levels. Only *NLRP1* in OV, *CASP8* in pheochromocytoma/paraganglioma (PCPG), glioblastoma (GBM), etc., showed a positive correlation between methylation and gene expression (*P*<0.05, **Figure 6B**). Kaplan-Meier survival analysis showed that *CHMP4C* and *IL6* hypermethylation in LGG and *IRF1* hypermethylation in UVM were associated with poor prognosis. Hypermethylation of *GSDMA* in ACC and hypermethylation of *GSDMB* in BLCA were associated with poor prognoses (**Figure 6C**).

### Relevant miRNA Regulation Analysis

miRNAs are non-coding RNAs that play important roles in the regulation of target mRNAs (20). To determine if miRNAs can regulate gene expression, VisNetwork was used to create regulatory miRNA networks. As shown in **Supplementary Figure 6**, miRNAs may regulate gene mRNA levels by targeting *TP63, CASP3, CASP6, CASP8*, and *CHMP7*. In particular, *IRF2* could be down-regulated by more miRNAs, including hsa-miR-133b, hsa-miR-498, hsa-miR-495-3p, hsa-miR-496, hsa-miR-3163, etc. In contrast, hsa-miR-383-5p and hsa-miR-183-5p could negatively regulate the expression of *IRF1*. These results suggest that gene expression may be regulated by miRNAs and may influence cancer progression.

### Pathway Activity Analysis

**Figure 7:**
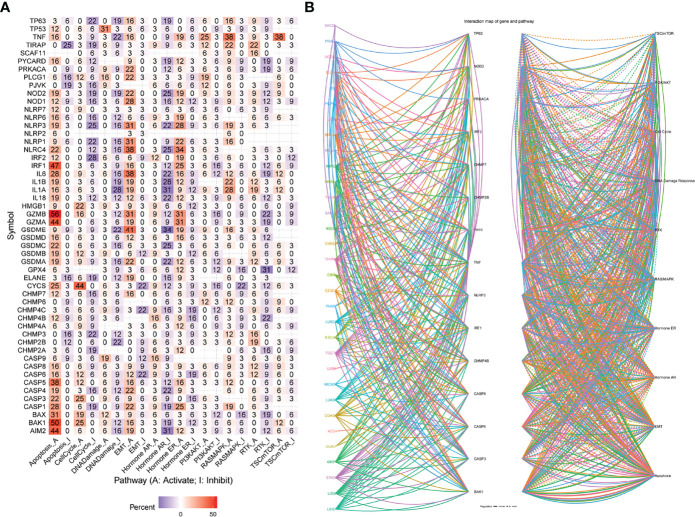
**(A)** Percentage of cancers in which mRNA levels of specific genes have potential pathway activity. **(B)** Network of the relationship between genes and pathways. Solid lines represent activation, dashed lines inhibition, and the color of the lines represents different cancer types.

From the heat map shown in **Figure 7A**, necroptosis genes were involved in TSC/mTOR, RTK, RAS/MAPK, PI3K/AKT, hormone ER, hormone AR, EMT, DNA damage response, cell cycle, and apoptosis pathways. *AIM2* is mainly involved in the activation of apoptosis, cell cycle, and inhibition of DNA damage response, hormone ER, PI3K/AKT; while *CHMP3* was mainly involved in inhibition of apoptosis, cell cycle, DNA damage response, hormone ER, as well as activation of RTK (**Figure 7B**). **Supplementary Figure 7** shows a sector diagram of molecular activation and repression signaling pathways associated with several necroptosis genes. These results suggest that necroptosis genes play an important role in regulating cancer-related metabolic pathways.

### Drug Sensitivity Analysis

Genomic alterations play an important role in carcinogenesis, disease progression, as well as resistance, and response to targeted therapy (21). To investigate the role of necroptosis genes in chemo- or targeted therapy, we integrated drug sensitivity data and gene expression profiles of cancer cell lines in the GDSC and CTRP databases. We performed a Pearson correlation analysis with a positive correlation meaning high gene expression is resistant to the drug, and *vice versa*. The results showed that in the GDSC database, the expression of *GZMB, NOD2, BAX, HMGB1, TP63, ELANE, NLRP3, GSDME*, and *CHMP4C* was negatively correlated with the drugs according to the IC_50_ values (**Supplementary Figure 8A**). In the CTRP database, *CASP3, CASP9, CHMP4A, CHMP7, CYCS, GZMA, HMGB1* were negatively correlated with drug sensitivity according to IC_50_ values (**Supplementary Figure 8B**). In conclusion, these results suggest that the above-mentioned genes with negative correlations may represent a new target for oncology drug therapy.

### Necroptosis Score

A necroptosis score for each tumor of the TCGA pan-cancer data was determined using the ssGSEA method of the R package “GSVA”. We found the highest DLBC levels in the tumor samples and the highest READ levels in the normal samples(**Supplementary Figure 9**). We compared the necroptosis scores of tumors with clinical staging and found a significant correlation between necroptosis scores and staging in diffuse large B-cell lymphoma (DLBC), ESCA, KIRC, HNSC, OV, pancreatic adenocarcinoma (PAAD), uterine corpus endometrial carcinoma (UCEC), and UVM (**Supplementary Figure 10**). Combining the necroptosis scores with the prognostic data of patients, it was found that high scores in UVM, LGG, and KIRC were associated with poor prognosis of the patients, while in skin cutaneous melanoma (SKCM), PCPG, and BRCA, high scores were associated with protective factors (**Supplementary Figure 11**). These results suggest a close association between necroptosis scores and patient outcomes. GSEA analysis showed that the scores were enriched in TNFA_SIGNALING_VIA_NFKB, INTERFERON_GAMMA_RESPONSE, IL6_JAK_STAT3_SIGNALING, and IL2_STAT5_SIGNALING (**Supplementary Figure 12**).

### High Necroptosis Scores Associated With Hot Tumor Microenvironments

**Figure 8:**
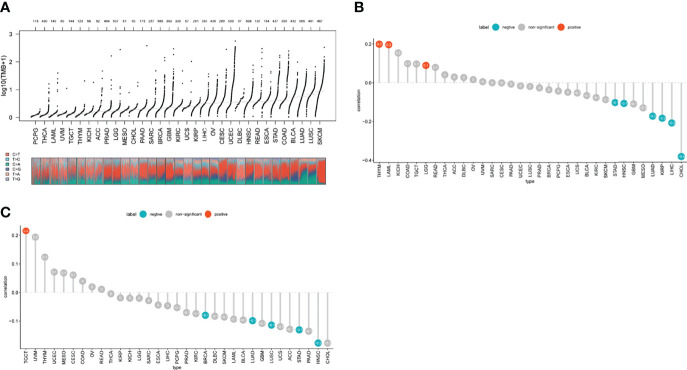
**(A)** TMB distribution of each tumor, with UCEC having the highest TMB **(B, C)** TME, and MSI analysis. Red points represent a positive correlation, and blue dots represent a negative orrelation. Numbers represent Pearson’s coefficient.

The tumor microenvironment (TME) consists of abnormal tumor vasculature, extracellular matrix components, endothelial cells, pericytes, tumor-associated fibroblasts, smooth muscle cells, and immune cells. Furthermore, the TME plays a crucial role in tumorigenesis, growth, and metastasis (22, 23). We used the R package “ESTIMATE” to calculate the stromal score, immune score, ESTIMATEScore, and tumor purity of tumor tissues. Furthermore, we also calculated the tumor mutational burden (TMB) as a biomarker to assess tumor immunotherapy benefits. TMB is defined as the total number of mutations per million bases in the exon coding region of a gene encoding a specific tumor cell protein, including insertions, substitutions, deletions, and other forms of mutations (24). It has been proven useful for predicting the specific treatment response of certain tumors. Moreover, due to the defective DNA mismatch repair during certain tumorigenesis, errors in microsatellite sequence replication cannot be detected in time, causing insertion or deletion of repetitive units and changes in microsatellite sequence length, which eventually lead to microsatellite instability (MSI) (25). Numerous clinical observations, retrospective studies, and meta-analyses have confirmed that MSI is closely related to tumor prognosis (26). We evaluated the relationship between the necroptosis score and the TME. We found that necroptosis was significantly correlated with TME (immune score and stromal score; **Supplementary Figure 13**). The necroptosis scores were also found to be closely related to tumor-related pathways (**Supplementary Figure 14**). The association between necroptosis and immune cell infiltration levels and types in 33 tumors was also assessed. A significant positive correlation was shown between the scores of most cancer types and the infiltration levels of natural killer, T follicular helper, T helper 1, CD8^+^ T, and dendritic cells (**Supplementary Figure 15**). **Figure 8A** demonstrates the TMB distribution of each tumor, with UCEC having the highest TMB. In addition, the necroptosis scores were positively correlated with the TMB of thymoma (THYM), acute myeloid leukemia (LAML), and with the MSI of TGCT (**Figures 8B, C**).

### Necroptosis Scores Associated With Therapeutic Responses in Multiple Cancer Types

**Figure 9:**
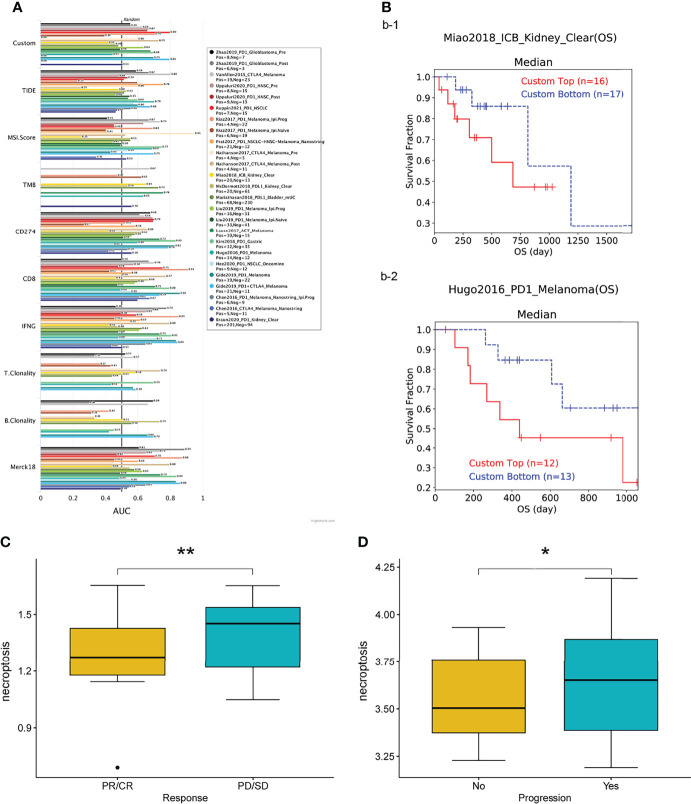
**(A)** Bar plot showing the biomarker relevance of necroptosis scores compared to standardized cancer immune evasion biomarkers in immune checkpoint blockade (ICB) sub-cohorts. The area under the receiver operating characteristic curve (AUC) was used to assess the predictive efficacy of test biomarkers for determining IBC response to TCGA for different cancer types. **(B–D)** Patients with high scores exhibit poorer prognostic outcomes after immunotherapy. CR, complete responses; PR, partial responses; PD, progressive disease; SD, stable disease. *P < 0.05, **P < 0.01.

We first assessed the relevance of biomarkers of necroptosis score, comparing them to standardized biomarkers based on their predictive power for OS outcomes in immune-checkpoint blockade (ICB) subgroups. Interestingly, we found 15 separate necroptosis scores in the 23 ICB subgroups with an area under the receiver operating characteristic curve (AUC) greater than 0.5 (**Figure 9A**). In turn., the necroptosis score exhibited a higher predictive value. Our results also suggest that higher scores are associated with worse PD1 and ICB outcomes in melanoma (Hugo2016_PD1), and KIRC (Miao2018_ICB) (**Figure 9B**). Patients with high scores have a worse prognosis after immunotherapy compared to patients with low scores and showed a worse prognostic outcome after immunotherapy. Patients with higher scores were also associated with a more progressive phase of cancer (**Figures 9C, D**).

### Experimental Verification

**Figure 10:**
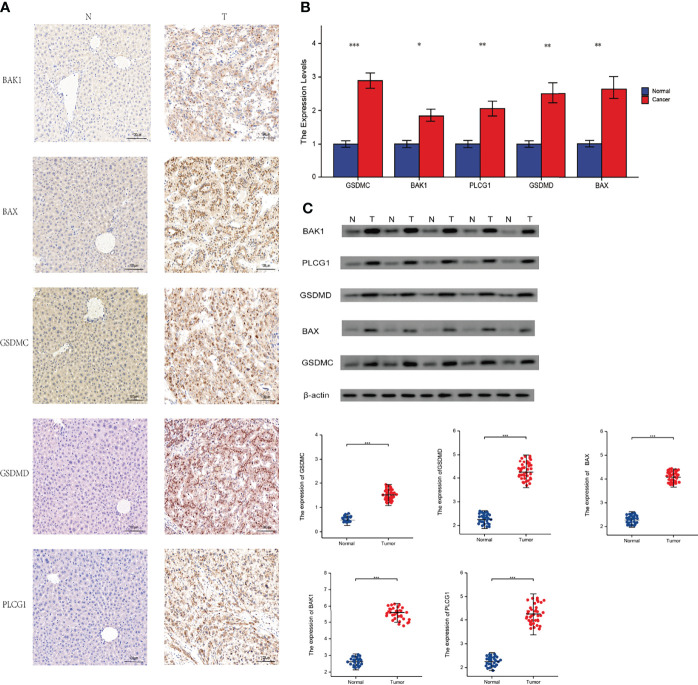
Experimental verification. T, tumor; N, normal. **(A)** Immunohistochemical stainings of 5 necroptosis genes in HCC. **(B)** Results of PCR analysis. **(C)** Results of Western Blot. *P < 0.05; **P < 0.01; ***P < 0.001

**Figure 11:**
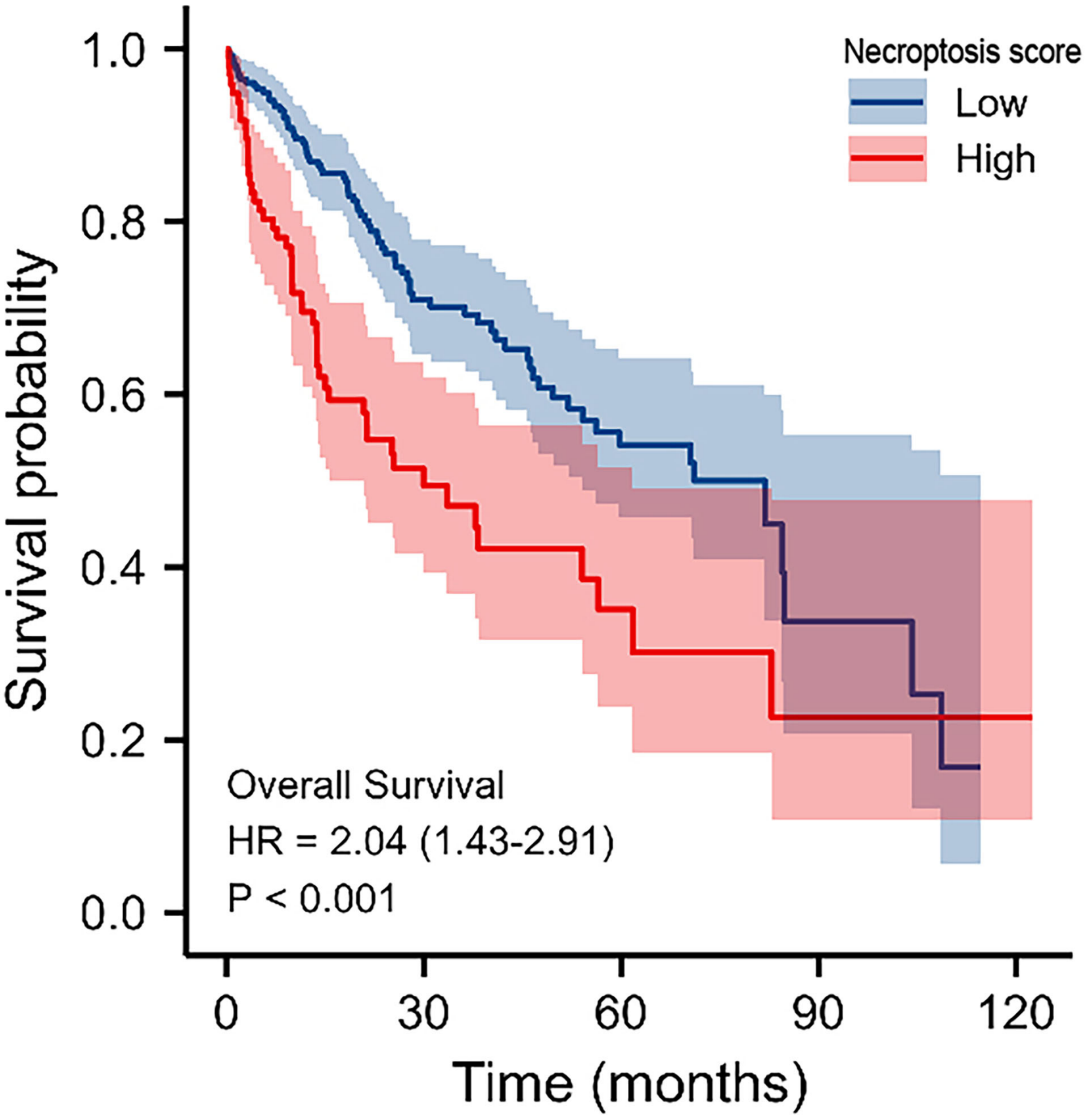
KM survival analysis of high‐ and low‐necroptosis score HCC samples.

After that, we validated our findings using HCC samples collected in our hospital as part of an independent cohort. In department of interventional radiotherapy, Shanghai Ninth People’s Hospital, paired adjacent non-HCC tissues and HCC tissues (n = 40 for each) were obtained from patients with HCC. Using histopathological methods, the diagnosis of HCC was confirmed. The study was approved by the Medical Ethics Committee of the Shanghai Ninth People’s Hospital and all individuals gave written informed consent. We selected the five most significantly differentially expressed genes for validation(BAK1, PLCG1, GSDMD, BAX, GSDMC). We first performed immunohistochemical (IHC) analysis and found that five genes were expressed at elevated levels in HCC tissue (**Figure 10A**). According to our previous study, we performed Real-time PCR analysis and Western blot (WB) analysis (27, 28). These results are in line with our findings using the databases(**Figures 10B, C**). We calculate the patient’s necroptosis score according to the ssGSEA method. According to the median levels of necroptosis score, HCC samples were divided into two groups based on high and low necroptosis scores. These results indicated that a high- necroptosis score patient’s prognosis was significantly worse than a low- necroptosis score patient’s (**Figure 11**).

## Discussion

In this study, we presented a complete and systematic description of genes in more than 9,000 samples from 33 types of cancer. Our results clarified the overall picture of necroptosis genes in cancer, revealing not only different potential mechanisms of necroptosis genes function in the context of cancer, but also common necroptosis genes associated with cancer pathways. The GTEx and TCGA databases were used to analyze the expression of necroptosis genes and their predictive value for tumor prognosis. We found aberrant gene expression in a variety of tumors and differential expression between tumor types that may reflect their unique underlying functions. We further investigated the relationship between genes and patient prognosis and identified necroptosis gene risk factors for patient prognosis in most tumors.

Cell death is considered to be an inevitable phenomenon of life. Cell death is often referred to as programmed cell death, which includes apoptosis, necroptosis and pyroptosis (29). Apoptosis is characterised by a cascade of Caspases, while necroptosis is characterised by activation of the RIPK1-RIPK3-MLKL pathway (30, 31). At the same time, there are similarities, differences and interactions between apoptosis, pyroptosis and necroptosis. Multiple modes of cell death may co-exist and interact with each other during the disease process. Previous articles have identified CASP8 as a “molecular switch” that controls apoptosis, pyroptosis and necroptosis (32). *GZMA* and *NLRP6* are also involved in the inflammatory cascade of apoptosis (33, 34).

Necroptosis plays an important role in maintaining homeostasis *in vivo*. It was reported that the expression of *RIP1* and *RIP3* was significantly reduced in colon cancer tissues compared with normal tissues, and the reduction of *RIP1* and *RIP3* weakened the response of tumor cells to necroptosis, which led to the survival of tumor cells (35). *RIP1/3* may produce antitumor metastatic results by modulating oxidative stress to kill metastatic tumor cells (36). In addition, necroptosis cells initiate acquired immunity by providing antigenic and inflammatory stimuli to dendritic cells, which in turn activate CD8^+^ T cells and anti-tumor immune responses (37). However, on the opposite spectrum, necroptosis may promote tumor progression. Strilic et al. found that tumor cells in humans and mice induce necroptosis in endothelial cells to promote tumor cell metastasis— an effect mediated by the binding of amyloid precursor proteins produced by tumor cells to the endothelial death receptor 6 (38). Necroptosis can promote tumor progression by activating an inflammatory response, while elevated *TNF-α* expression in the TME is characteristic of many malignancies and associated with poor prognosis (39). Thus, necroptosis inhibits tumorigenesis and progression by killing tumor cells but also promotes tumorigenesis *via* a tumor immunosuppressive effect and cytokine-induced inflammatory response from necrotic cells. This indicates necroptosis as a promising target for tumor therapy though its role in tumors still needs to be studied in depth. The study of necroptosis is therefore of great importance.

From our genetic analysis, we found a high frequency of necroptosis genes in CNVs. Our analysis confirms that CNVs are positively correlated with necroptosis genes expression. This demonstrates that CNVs can influence necroptosis genes expression and in turn promote tumorigenesis. Analysis of epigenetic modifications of individual necroptosis genes showed that aberrant hypermethylation of necroptosis genes mediated their down-regulation and was associated with poor prognosis in several cancers. Hypermethylation and survival analysis suggested that hypermethylated *CHMP4B* and *NOD1* genes may play a driver gene role in THYM and UVM, respectively. A large number of studies have reported that hypermethylation is associated with poor prognosis in various cancers (40, 41). Thus, the above analysis suggests that genetic and epigenetic modifications of necroptosis genes can lead to dysfunction and are often involved in tumorigenesis. Given that necroptosis genes play an important role in cancer, it is crucial to identify their regulatory molecules. With the help of the miRNA-mRNA interaction network, we found that *TP63, CASP3, CASP6, CASP8, CHMP7* can be regulated by miRNAs. Shu et al. reported that necroptosis could be regulated by miR-15b-5p (42). Our results suggest that necroptosis genes can be influenced by other miRNAs that allowed us to identify therapeutic targets. We also found that necroptosis genes are associated with tumor-associated pathways (DNA damage response, cell cycle, and apoptosis pathways) in a variety of tumors. Furthermore, by constructing GSVA scores, we found that the scores correlated with patient prognosis, clinical stage and the efficacy of immunotherapy, which further suggests an important role for necroptosis in tumors.

Our drug sensitivity analysis identified potential drugs (GSK-J4, belinostat) that may target necroptosis genes. Therefore, we anticipate that necroptosis genes targeting will become an ideal approach in cancer treatment. However, the mechanisms underlying the effects of these drugs on necroptosis genes expression and cancer progression need further investigation.

## Conclusion

In this study, we compared necroptosis gene expression in tumors and corresponding normal tissues. Our study showed that genomic variation in the SNV and CNV genomes had an impact on mRNA levels and survival. In addition, we used multiple databases to predict the effect of necroptosis genes on immunotherapy efficacy. We also found that necroptosis genes can be regulated by miRNAs and identified necroptosis genes for targeted drug therapy. Thus, our study provides insight into potential tumor immunotherapy targets related to necroptosis.

## Data Availability Statement

The datasets presented in this study can be found in online repositories. The names of the repository/repositories and accession number(s) can be found in the article/**Supplementary Material**.

## Ethics Statement

The study was reviewed and approved by the Medical Ethics Committee of the Shanghai Ninth People’s Hospital and all individuals gave written informed consent.

## Author Contributions

X-TY and X-YL carried out experiments, X-YL, HL, L-YZ, Y-HS, Y-CS, J-XY, J-BW, LZ, DW, M-ZW, and ZW wrote the manuscript, Y-CS, J-XY, J-BW, LZ, DW, M-ZW, and ZW are responsible for samples and clinical data collections. X-TY performed manuscript review. All authors contributed to the article and approved the submitted version.

## Funding

This study received Fundamental research program funding of Ninth People’s Hospital affiliated to Shanghai Jiao Tong university School of Medicine (No. JYZZ076), Clinical Research Program of Ninth People’s Hospital, Shanghai Jiao Tong University School of Medicine (No. JYLJ201801, JYLJ201911), the China Postdoctoral Science Foundation (No. 2017M611585) and the National Natural Science Foundation of China (No. 81871458).

## Conflict of Interest

The authors declare that the research was conducted in the absence of any commercial or financial relationships that could be construed as a potential conflict of interest.

## Publisher’s Note

All claims expressed in this article are solely those of the authors and do not necessarily represent those of their affiliated organizations, or those of the publisher, the editors and the reviewers. Any product that may be evaluated in this article, or claim that may be made by its manufacturer, is not guaranteed or endorsed by the publisher.

## Supplementary Material

Supplementary Figure 1 The heat map shows the expression profiles of the necroptosis -related genes in the GTEx dataset.

Supplementary Figure 2 Changes in mRNA levels in different cancer subtypes.

Supplementary Figure 3
**(A)** Positive relationship between necroptosis genes. **(B)** Genomic mutations for necroptosis genes.

Supplementary Figure 4 SNV percentage heatmap.

Supplementary Figure 5 Survival difference between mutant and WT. The size of the dots represents the significance of the influence on survival for each cancer type, and the statistical significance of differences was determined by cox regression analysis. Red dots represent worse survival and blue dots represent the opposite.

Supplementary Figure 6 miRNA regulation network. A node represents a miRNA or gene; an edge represents regulation of miRNA to gene.

Supplementary Figure 7 Global percentage of cancers in which a gene affects the pathway of cancer types.

Supplementary Figure 8 Drug sensitivity analysis: **(A)** The gene set drug sensitivity analysis from Genomics of Drug Sensitivity in Cancer (GDSC) IC50 drug data. **(B)** The gene set drug sensitivity analysis from Cancer Therapeutics Response Portal (CTRP) IC50 drug data. The Pearson’s correlation indicates the correlation between gene expression and drugs sensitivity. Blue bubbles represented negative correlations, and red bubbles represented positive correlations; the deeper the color, the higher the correlation. The bubble size was positively correlated with the FDR significance. The black outline indicates an FDR < 0.05.

Supplementary Figure 9 GSVA score in selected cancer types.

Supplementary Figure 10 GSVA score in stages of selected cancer types.

Supplementary Figure 11 Survival between high and low GSVA score groups. The color represents the hazard ratio (HR), and the size represents the P-value.

Supplementary Figure 12 Enrichment scores of gene set in the selected cancers.

Supplementary Figure 13 Analysis of the tumor microenvironment. Colors represent positive and negative correlations. Numbers represent Pearson’s coefficient.

Supplementary Figure 14 The association between GSVA score and activity of cancer-related pathways in selected cancers. *: P value ≤ 0.05; ^#^FDR ≤ 0.05.

Supplementary Figure 15 The association between GSVA score and immune cell type in selected cancers. *P value ≤ 0.05; ^#^FDR ≤ 0.05.

Supplementary Table 1 The list of abbreviations.

Supplementary Table 2 Univariate analysis and multivariate analysis.
