# Supplementary figures and images for: Clinical Implications of Necroptosis Genes Expression for Cancer Immunity and Prognosis: A Pan-Cancer Analysis
Source: Front Immunol. 2022 Jun 20;13:882216. doi: 10.3389/fimmu.2022.882216 (PMC9251086; doi:10.3389/fimmu.2022.882216)

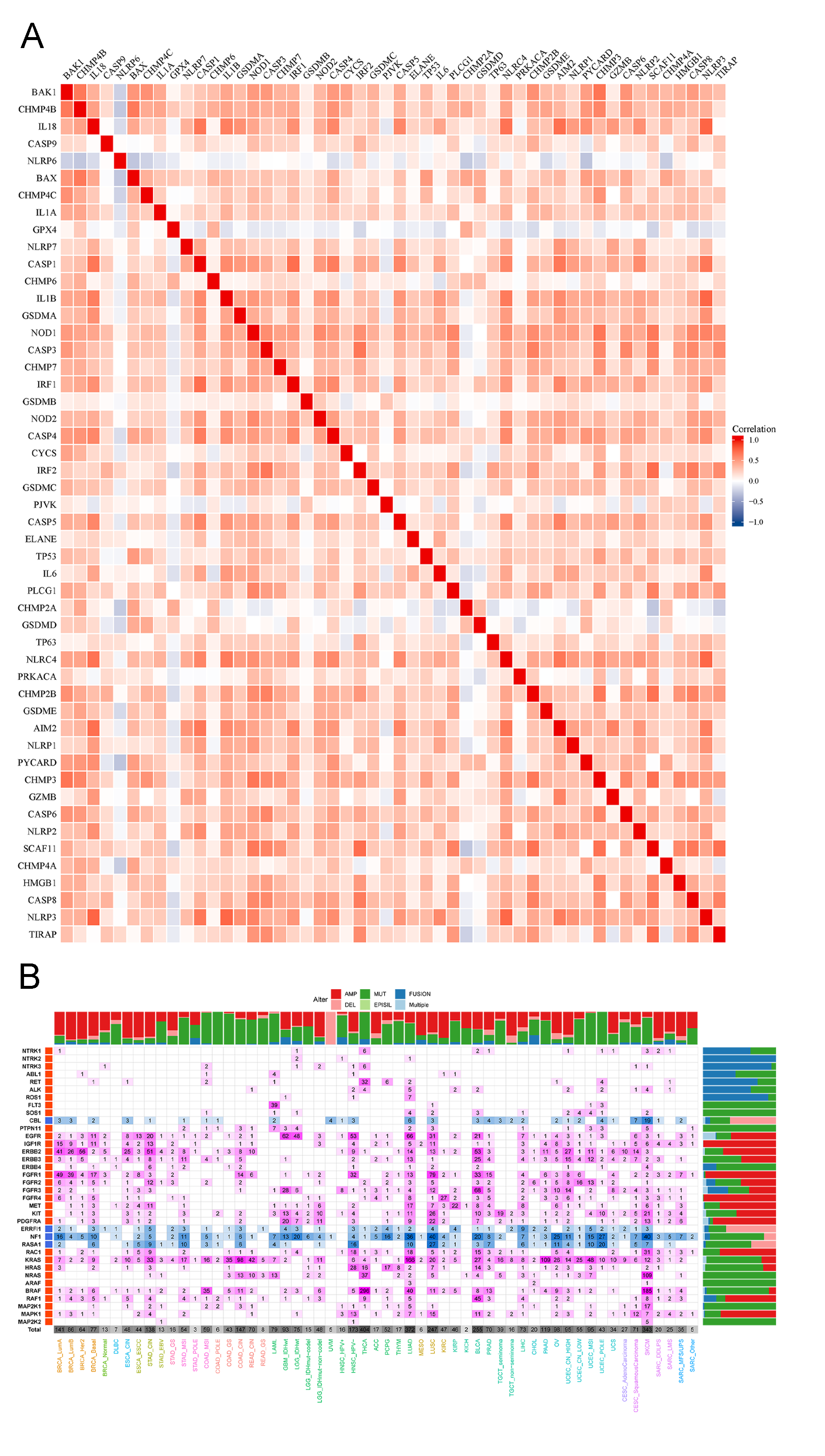

Supplement: Supplementary Figure 3 — (A) Positive relationship between necroptosis genes. (B) Genomic mutations for necroptosis genes. [file Image_3.tif]

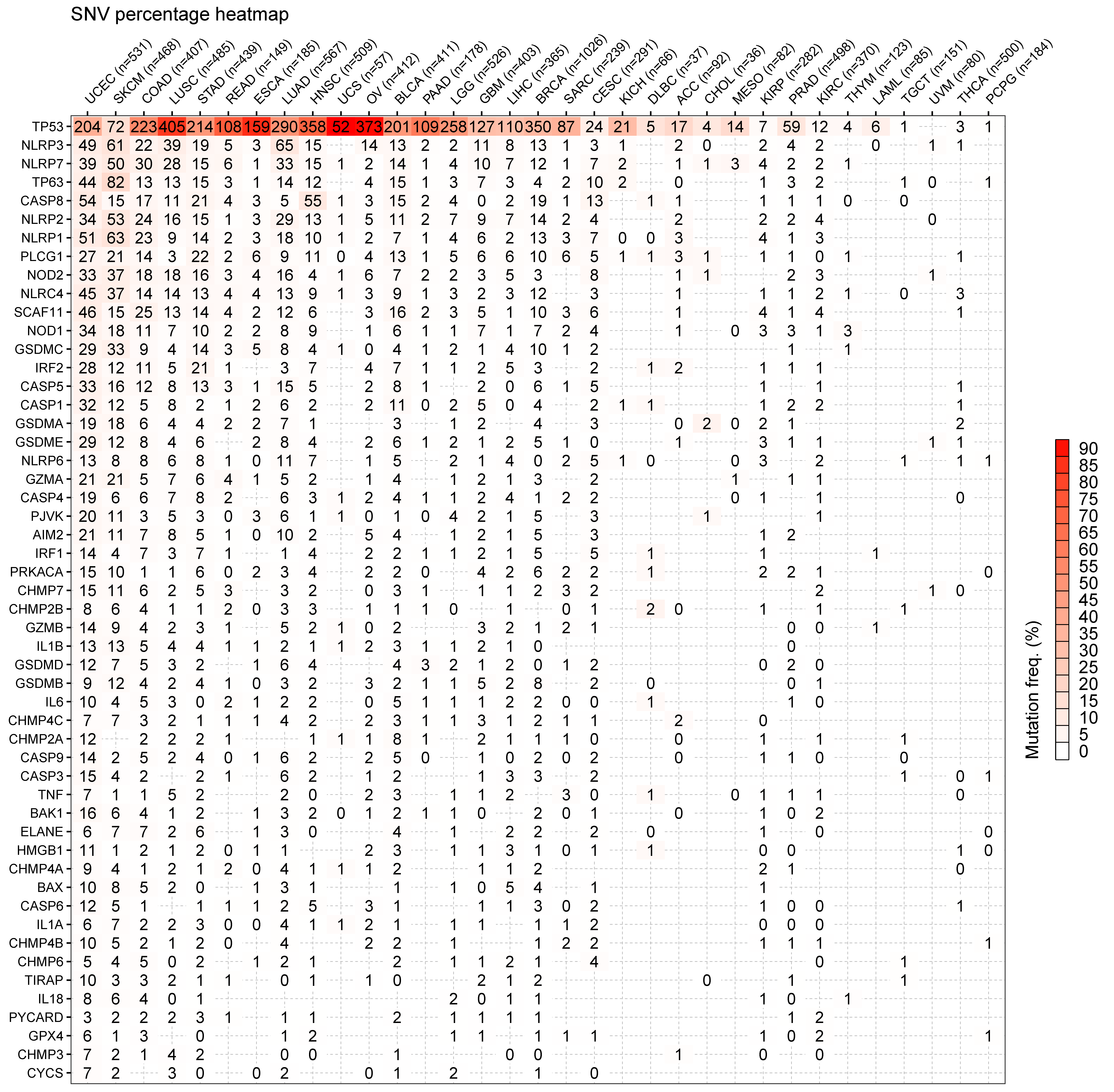

Supplement: Supplementary Figure 4 — SNV percentage heatmap. [file Image_4.tif]

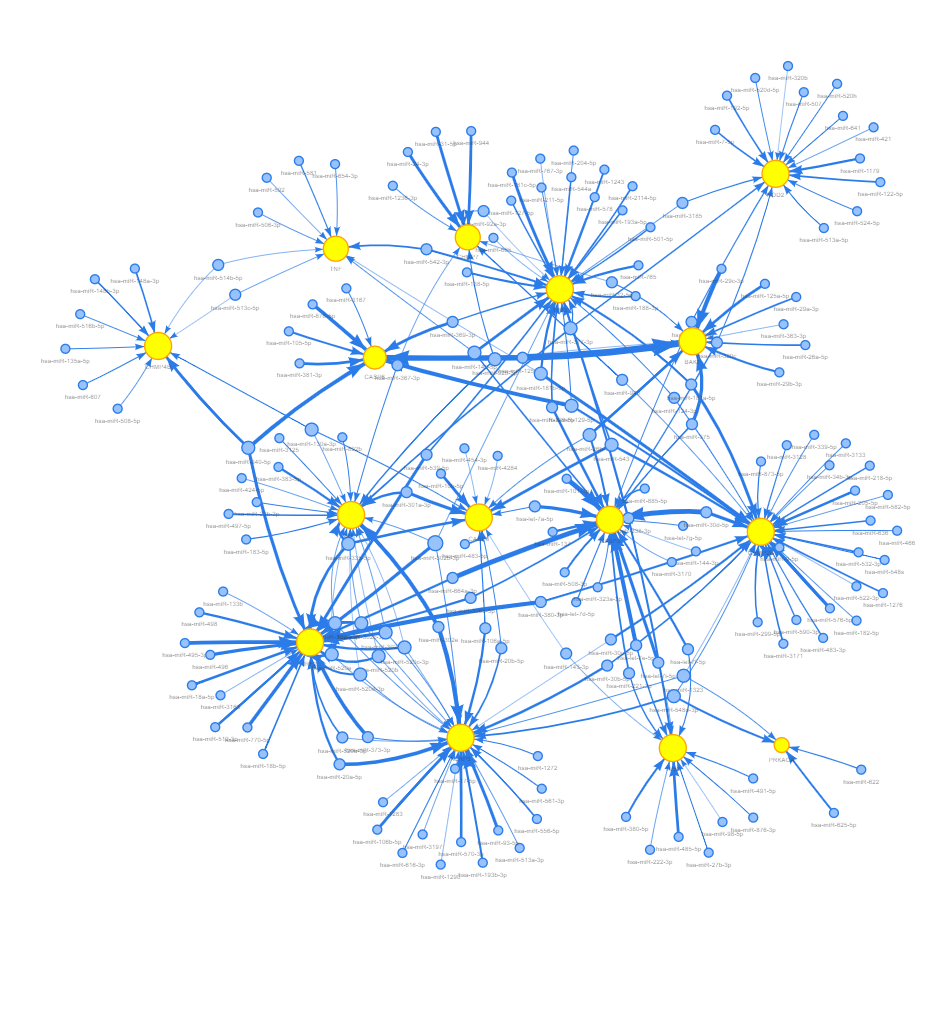

Supplement: Supplementary Figure 6 — miRNA regulation network. A node represents a miRNA or gene; an edge represents regulation of miRNA to gene. [file Image_6.tif]

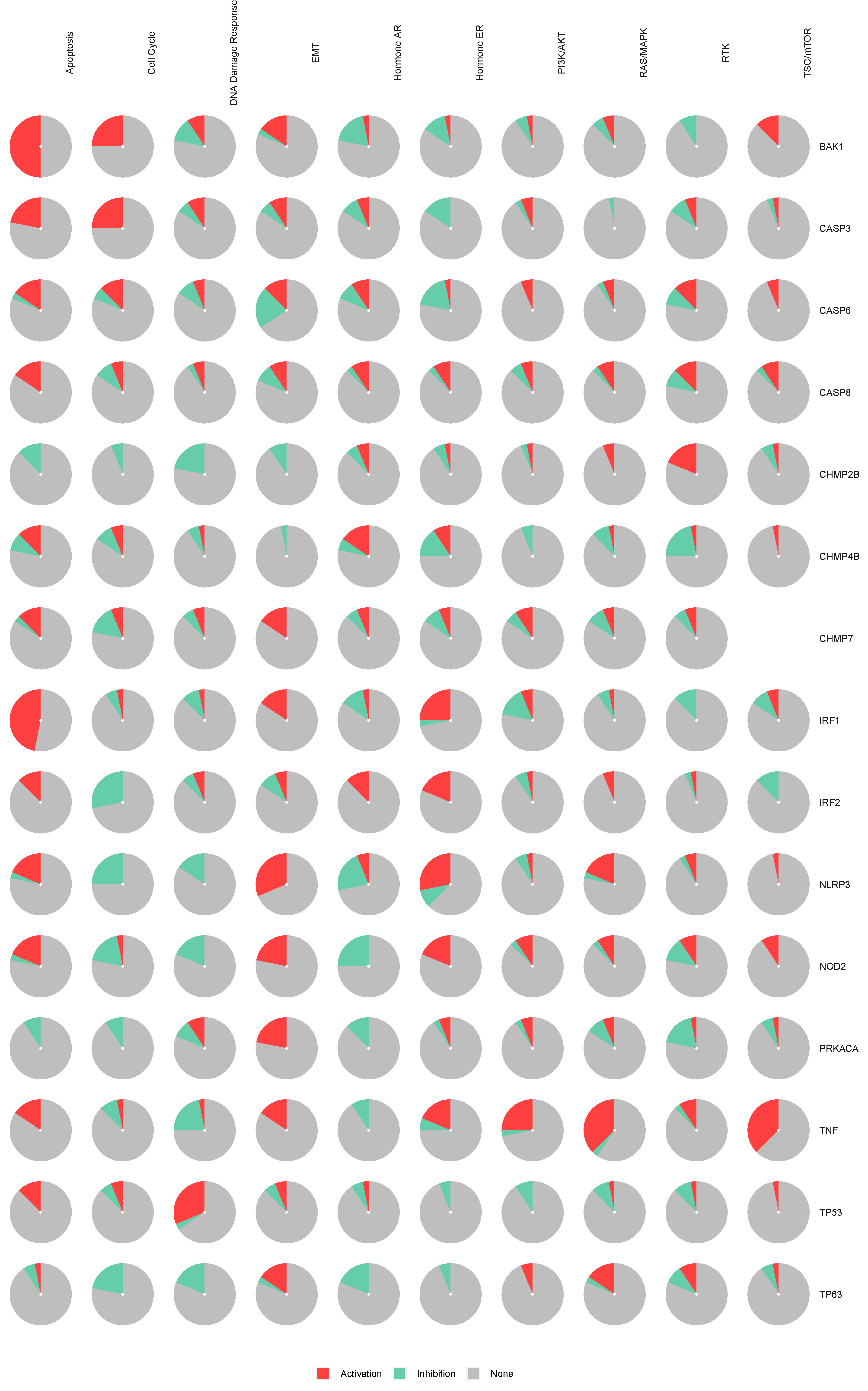

Supplement: Supplementary Figure 7 — Global percentage of cancers in which a gene affects the pathway of cancer types. [file Image_7.tif]

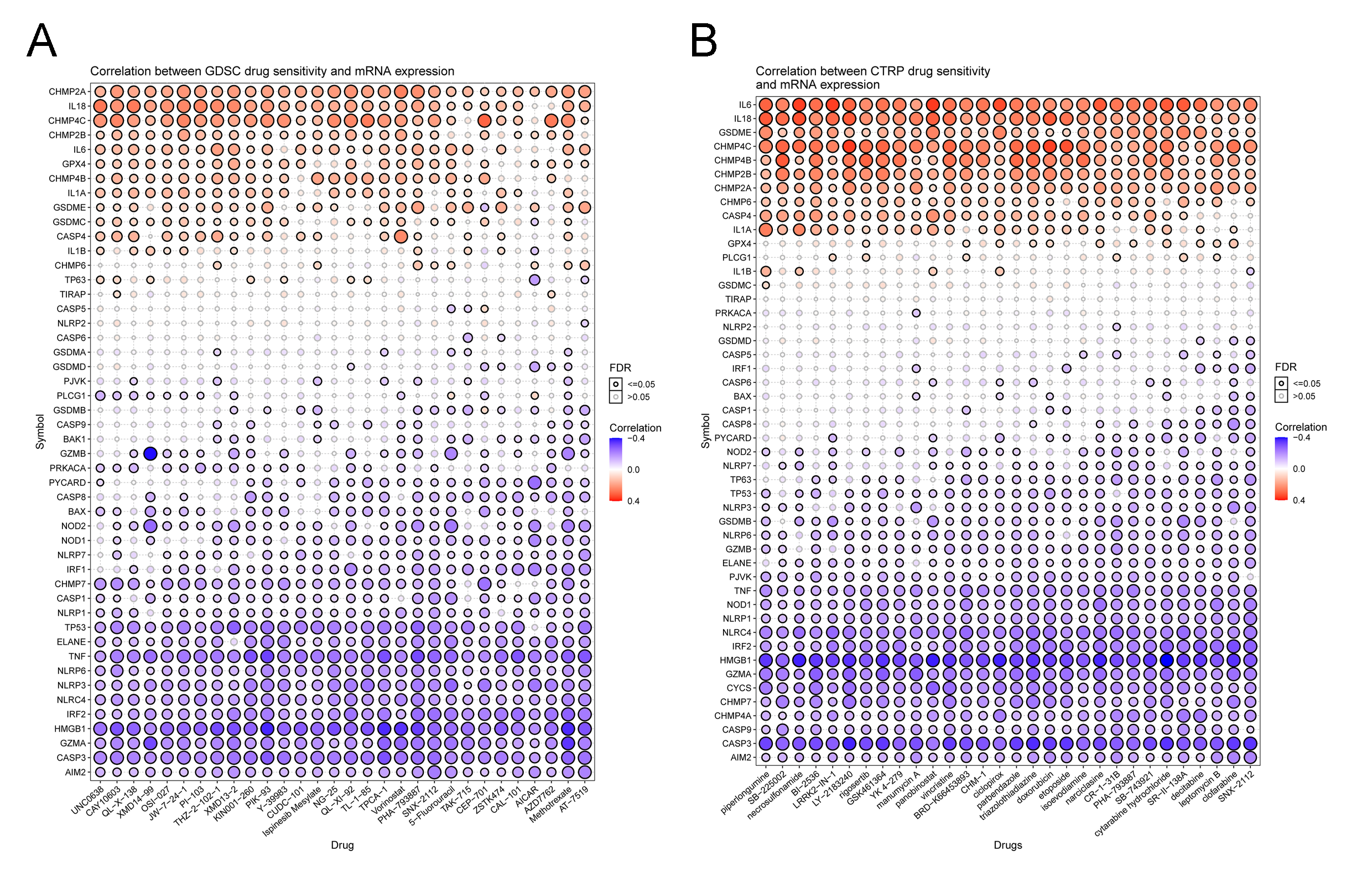

Supplement: Supplementary Figure 8 — Drug sensitivity analysis: (A) The gene set drug sensitivity analysis from Genomics of Drug Sensitivity in Cancer (GDSC) IC50 drug data. (B) The gene set drug sensitivity analysis from Cancer Therapeutics Response Portal (CTRP) IC50 drug data. The Pearson’s correlation indicates the correlation between gene expression and drugs sensitivity. Blue bubbles represented negative correlations, and red bubbles represented positive correlations; the deeper the color, the higher the correlation. The bubble size was positively correlated with the FDR significance. The black outline indicates an FDR < 0.05. [file Image_8.tif]

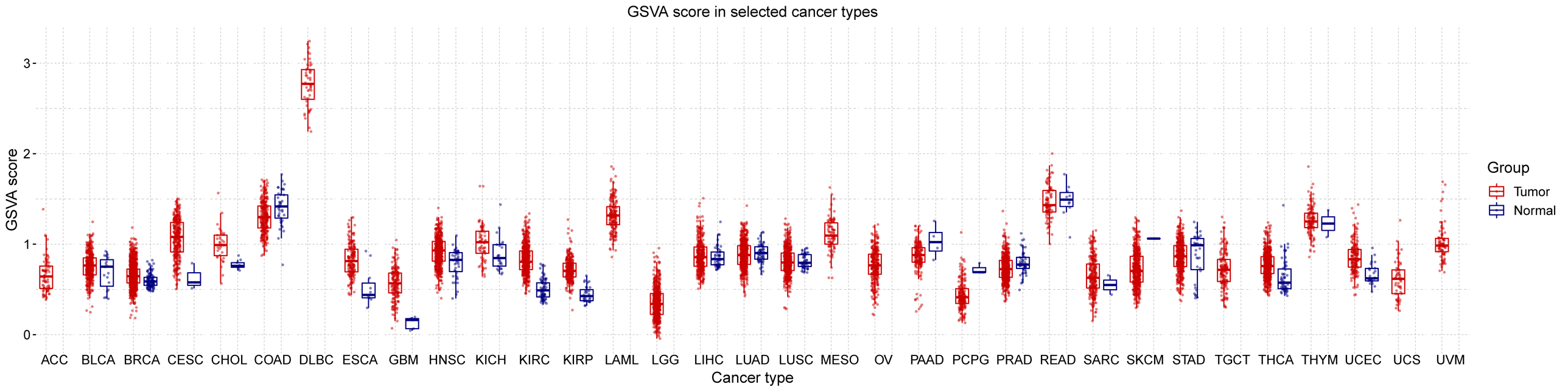

Supplement: Supplementary Figure 9 — GSVA score in selected cancer types. [file Image_9.tif]

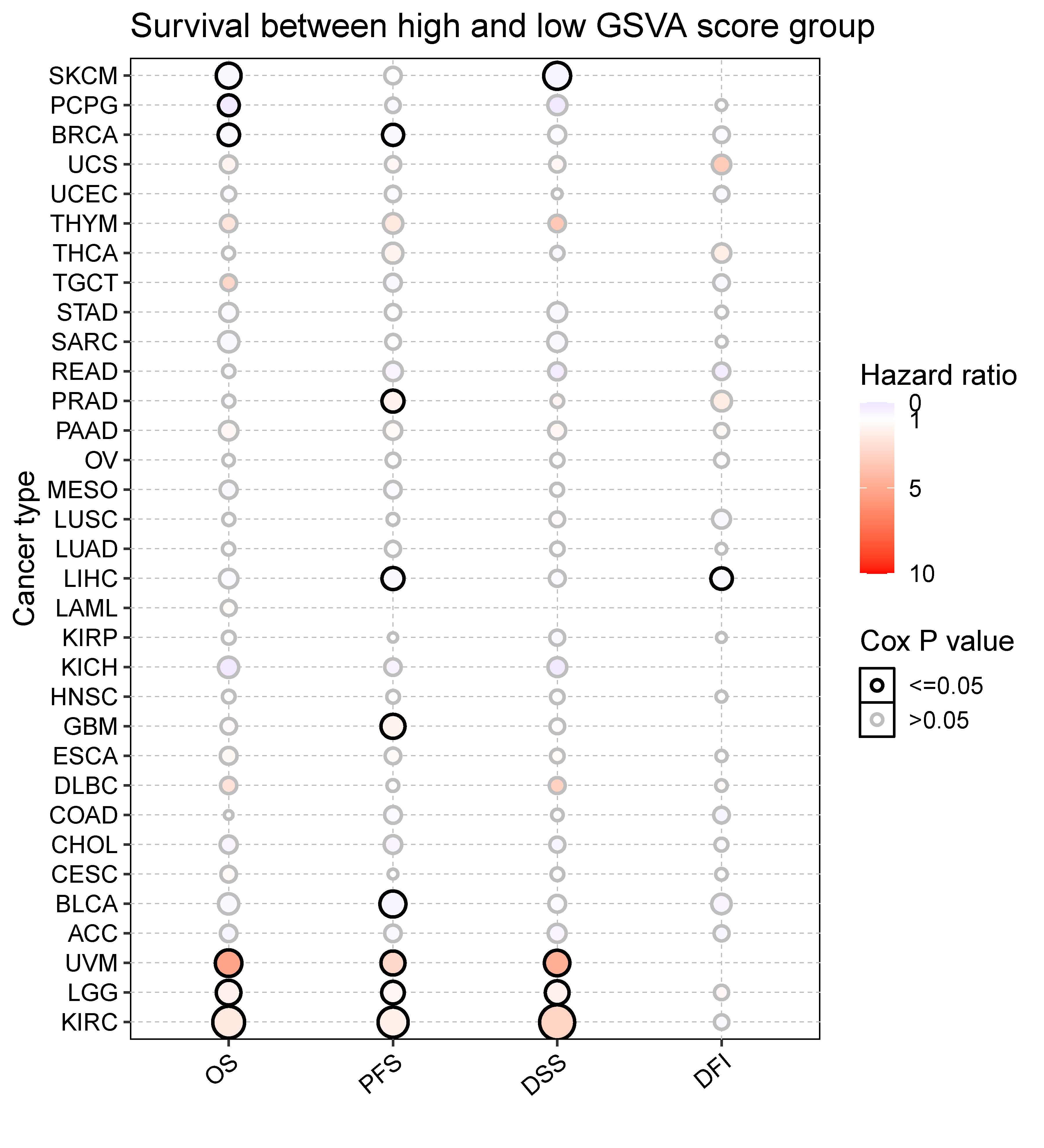

Supplement: Supplementary Figure 11 — Survival between high and low GSVA score groups. The color represents the hazard ratio (HR), and the size represents the P-value. [file Image_11.tif]

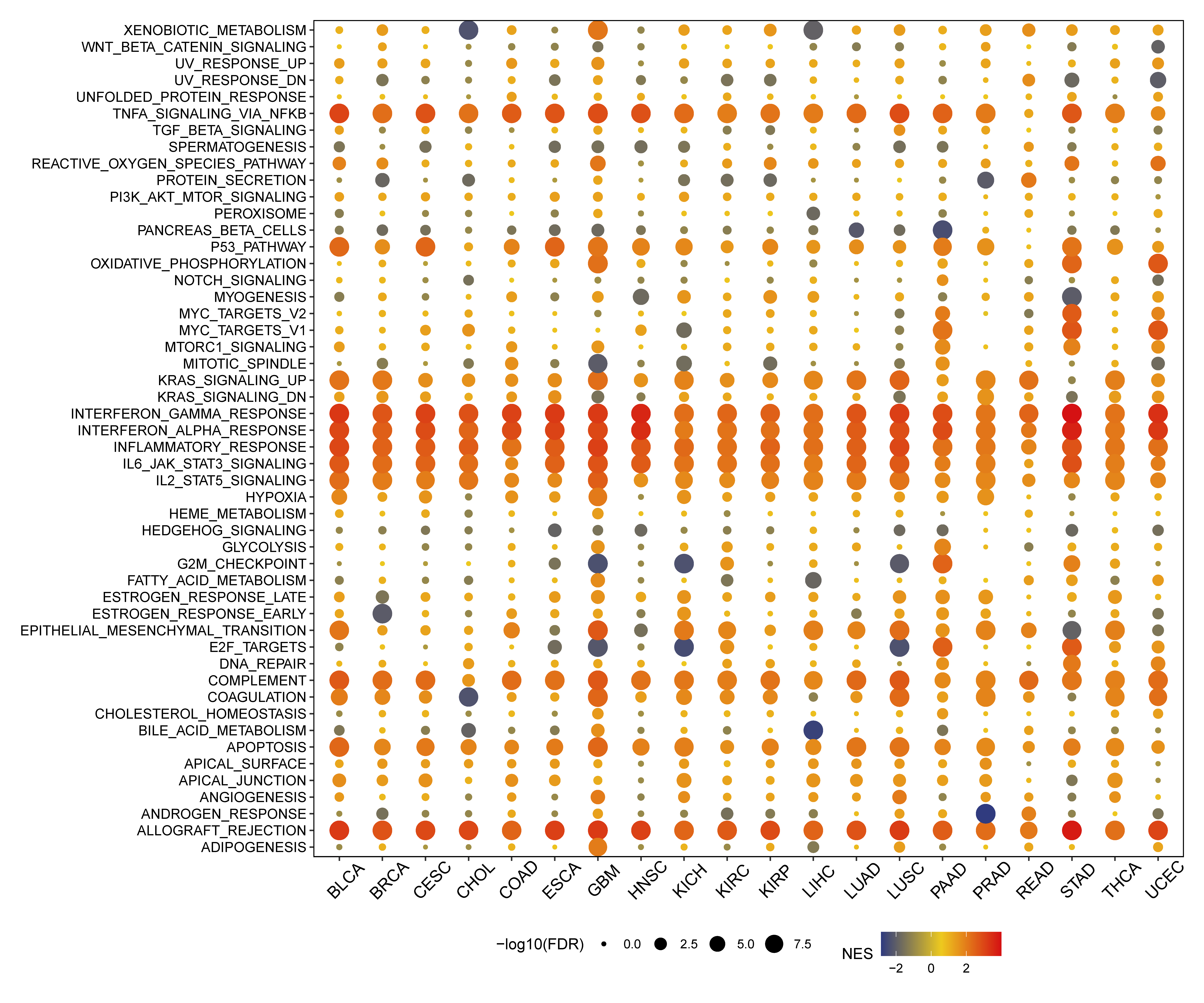

Supplement: Supplementary Figure 12 — Enrichment scores of gene set in the selected cancers. [file Image_12.tif]

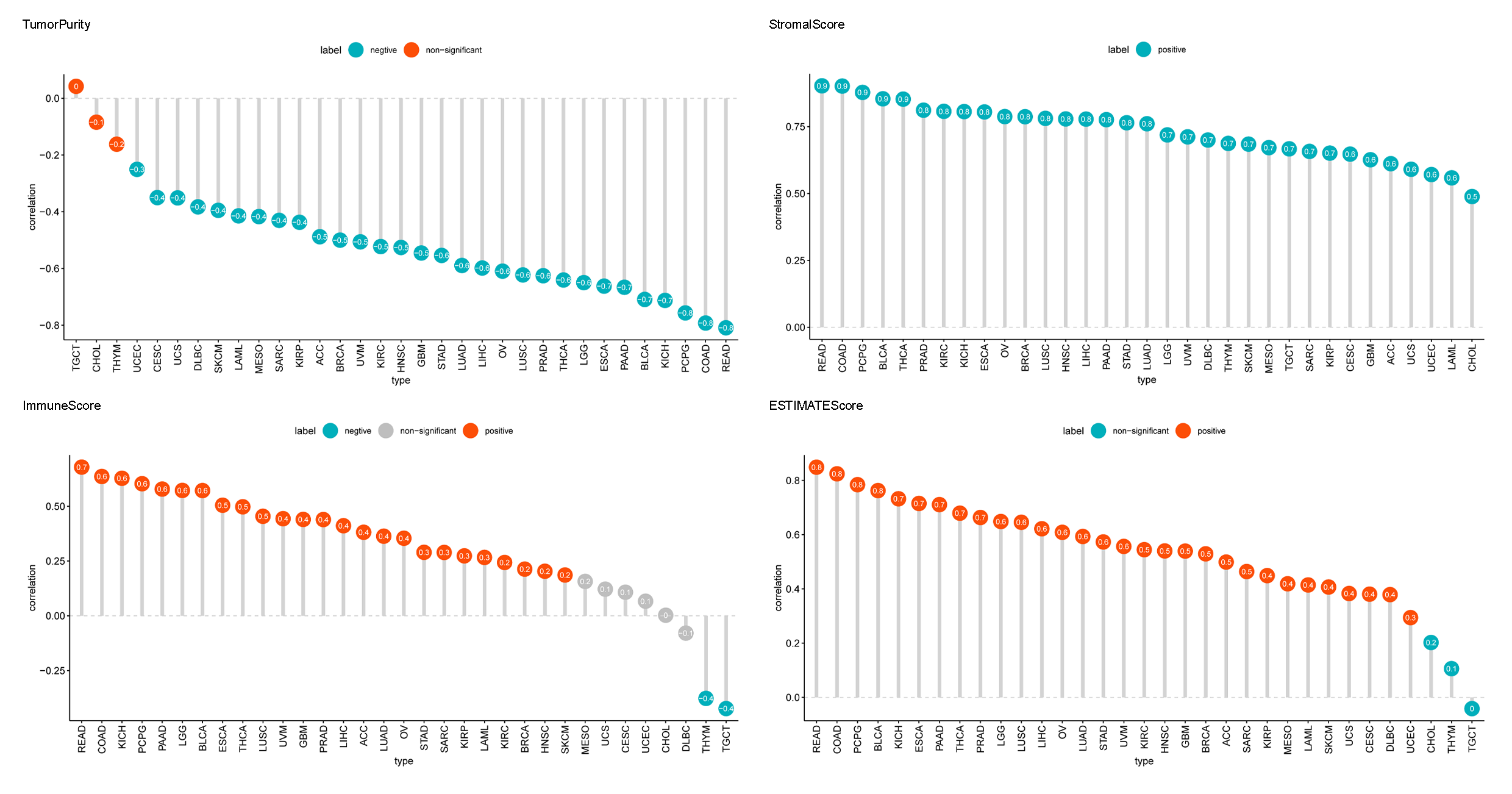

Supplement: Supplementary Figure 13 — Analysis of the tumor microenvironment. Colors represent positive and negative correlations. Numbers represent Pearson’s coefficient. [file Image_13.tif]

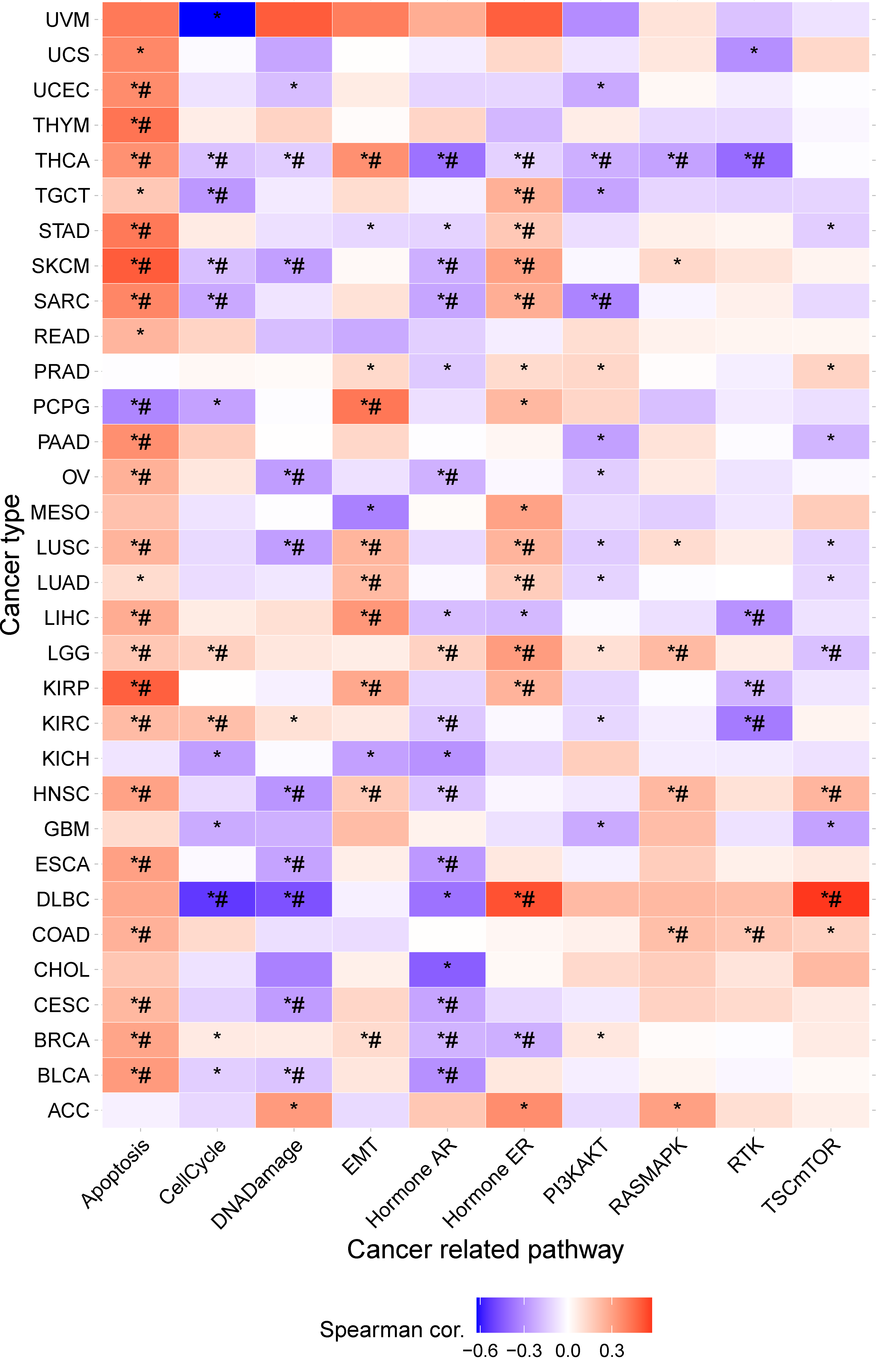

Supplement: Supplementary Figure 14 — The association between GSVA score and activity of cancer-related pathways in selected cancers. *: P value ≤ 0.05; #FDR ≤ 0.05. [file Image_14.tif]

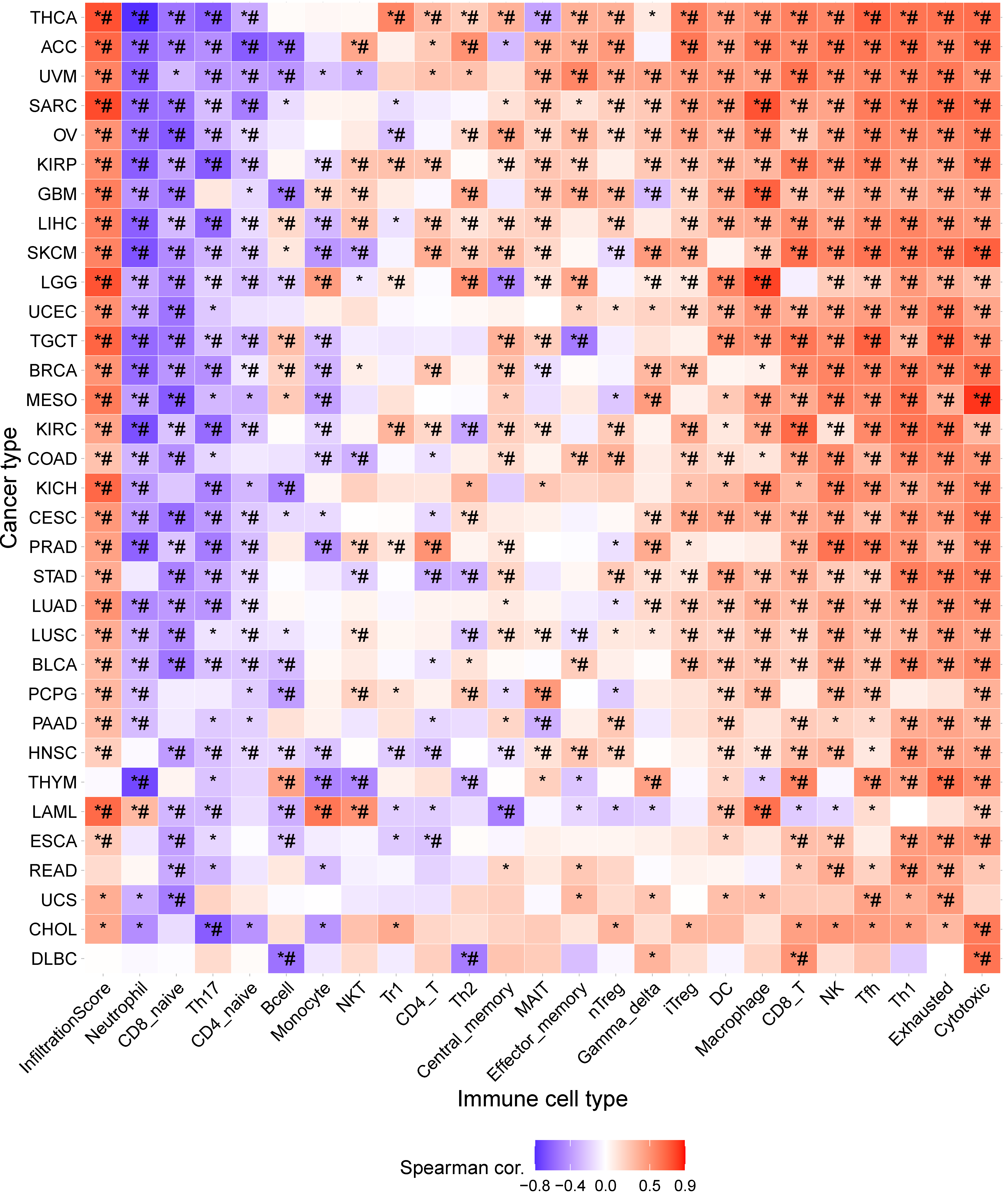

Supplement: Supplementary Figure 15 — The association between GSVA score and immune cell type in selected cancers. *P value ≤ 0.05; #FDR ≤ 0.05. [file Image_15.tif]
