# Supplementary material for: Clinical Implications of Necroptosis Genes Expression for Cancer Immunity and Prognosis: A Pan-Cancer Analysis
Source: Front Immunol. 2022 Jun 20;13:882216. doi: 10.3389/fimmu.2022.882216 (PMC9251086; doi:10.3389/fimmu.2022.882216)
Supplement: Supplementary Table 1 — The list of abbreviations. [file Table_1.docx]

**Abbreviations**

ACC, adenoid cystic carcinoma

AR, androgen receptor

AUC, area under the receiver operating characteristic curve

BLCA, bladder urothelial carcinoma

BRCA, breast cancer

CHOL, cholangiocarcinoma

CNV, copy number variation

COAD, colon adenocarcinoma

CTRP, Cancer Therapeutics Response Portal

DFS, disease-free survival

DLBC, diffuse large B-cell lymphoma

DSS, disease-specific survival

EMT, epithelial-mesenchymal transition

ER, estrogen receptor

ESCA, esophageal carcinoma

FC, fold-change

FDR, false discovery rate

GBM, glioblastoma

GDSC, Genomics of Drug Sensitivity in Cancer

GTEx, Genotype-Tissue Expression Portal

HR, hazard ratio

IC_50_, half-maximal inhibitory concentration

ICB, immune-checkpoint blockade

KICH, kidney chromophobe

KIRC, renal clear cell carcinoma

KIRP, kidney renal papillary cell carcinoma

LAML, acute myeloid leukemia

LGGs, low-grade gliomas

LICH, liver hepatocellular carcinoma

LUAD, lung adenocarcinoma

LUSC, lung squamous cell carcinoma

MESO, mesothelioma

miRNA, microRNA

MSI, microsatellite instability

OS, overall survival

OS, overall survival

OV, ovarian cancer

PAAD, pancreatic adenocarcinoma

PAS, pathway activity score

PCPG, pheochromocytoma/paraganglioma

PFS, progression-free survival

PRAD, prostate adenocarcinoma

READ, rectum adenocarcinoma

RPPA, reverse-phase protein array

SKCM, skin cutaneous melanoma

SNV, single-nucleotide variation

TCGA, The Cancer Genome Atlas

TGCT, Tenosynovial giant cell tumor

THCA, thyroid cancer

THYM, thymoma

Ti, transitions

TMB, tumor mutational burden

TME, tumor microenvironment

TPM, transcripts per million

Tv, transversions

UCEC, uterine corpus endometrial carcinoma

UCS, uterine carcinosarcomas

UVM, uveal melanoma
