# Supplementary material for: Clinical Implications of Necroptosis Genes Expression for Cancer Immunity and Prognosis: A Pan-Cancer Analysis
Source: Front Immunol. 2022 Jun 20;13:882216. doi: 10.3389/fimmu.2022.882216 (PMC9251086; doi:10.3389/fimmu.2022.882216)
Supplement: Supplementary Table 2 — Univariate analysis and multivariate analysis. [file Table_2.docx]

| Characteristics | Total(N) | Univariate analysis | |  | Multivariate analysis | |
| --- | --- | --- | --- | --- | --- | --- |
|  |  | Hazard ratio (95% CI) | P value |  | Hazard ratio (95% CI) | P value |
| WHO grade | 466 |  |  |  |  |  |
| G2 | 223 | Reference |  |  |  |  |
| G3 | 243 | 3.059 (2.046-4.573) | **<0.001** |  | 2.145 (1.394-3.300) | **<0.001** |
| CASP9 | 527 |  |  |  |  |  |
| Low | 264 | Reference |  |  |  |  |
| High | 263 | 0.595 (0.423-0.837) | **0.003** |  | 0.618 (0.448-0.852) | **<0.001** |
| Age | 527 |  |  |  |  |  |
| <=40 | 264 | Reference |  |  |  |  |
| >40 | 263 | 2.889 (2.009-4.155) | **<0.001** |  | 3.138 (2.043-4.820) | **<0.001** |
| Gender | 527 |  |  |  |  |  |
| Female | 238 | Reference |  |  |  |  |
| Male | 289 | 1.124 (0.800-1.580) | 0.499 |  |  |  |
| IDH status | 524 |  |  |  |  |  |
| WT | 97 | Reference |  |  |  |  |
| Mut | 427 | 0.186 (0.130-0.265) | **<0.001** |  | 0.395 (0.228-0.682) | **<0.001** |
| 1p/19q codeletion | 527 |  |  |  |  |  |
| codel | 170 | Reference |  |  |  |  |
| non-codel | 357 | 2.493 (1.590-3.910) | **<0.001** |  | 2.174 (1.249-3.785) | **0.006** |

**Univariate analysis and multivariate analysis for *CASP9* in OS of LGG**

**Univariate analysis and multivariate analysis for *PLCG1* in OS of LGG**

| Characteristics | Total(N) | Univariate analysis | |  | Multivariate analysis | |
| --- | --- | --- | --- | --- | --- | --- |
|  |  | Hazard ratio (95% CI) | P value |  | Hazard ratio (95% CI) | P value |
| WHO grade | 466 |  |  |  |  |  |
| G2 | 223 | Reference |  |  |  |  |
| G3 | 243 | 3.059 (2.046-4.573) | **<0.001** |  | 1.852 (1.192-2.878) | **0.006** |
| PLCG1 | 527 |  |  |  |  |  |
| Low | 263 | Reference |  |  |  |  |
| High | 264 | 3.364 (2.284-4.954) | **<0.001** |  | 1.884 (1.221-2.908) | **0.004** |
| Age | 527 |  |  |  |  |  |
| <=40 | 264 | Reference |  |  |  |  |
| >40 | 263 | 2.889 (2.009-4.155) | **<0.001** |  | 2.914 (1.898-4.474) | **<0.001** |
| Gender | 527 |  |  |  |  |  |
| Female | 238 | Reference |  |  |  |  |
| Male | 289 | 1.124 (0.800-1.580) | 0.499 |  |  |  |
| IDH status | 524 |  |  |  |  |  |
| WT | 97 | Reference |  |  |  |  |
| Mut | 427 | 0.186 (0.130-0.265) | **<0.001** |  | 0.358 (0.227-0.565) | **<0.001** |
| 1p/19q codeletion | 527 |  |  |  |  |  |
| codel | 170 | Reference |  |  |  |  |
| non-codel | 357 | 2.493 (1.590-3.910) | **<0.001** |  | 1.796 (1.070-3.014) | **0.027** |

**Univariate analysis and multivariate analysis for GSDMC in OS of LGG**

| Characteristics | Total(N) | Univariate analysis | |  | Multivariate analysis | |
| --- | --- | --- | --- | --- | --- | --- |
|  |  | Hazard ratio (95% CI) | P value |  | Hazard ratio (95% CI) | P value |
| WHO grade | 466 |  |  |  |  |  |
| G2 | 223 | Reference |  |  |  |  |
| G3 | 243 | 3.059 (2.046-4.573) | **<0.001** |  | 2.118 (1.373-3.266) | **<0.001** |
| GSDMC | 527 |  |  |  |  |  |
| Low | 263 | Reference |  |  |  |  |
| High | 264 | 0.628 (0.442-0.890) | **0.009** |  | 0.946 (0.615-1.454) | 0.800 |
| Age | 527 |  |  |  |  |  |
| <=40 | 264 | Reference |  |  |  |  |
| >40 | 263 | 2.889 (2.009-4.155) | **<0.001** |  | 3.058 (1.996-4.685) | **<0.001** |
| Gender | 527 |  |  |  |  |  |
| Female | 238 | Reference |  |  |  |  |
| Male | 289 | 1.124 (0.800-1.580) | 0.499 |  |  |  |
| IDH status | 524 |  |  |  |  |  |
| WT | 97 | Reference |  |  |  |  |
| Mut | 427 | 0.186 (0.130-0.265) | **<0.001** |  | 0.323 (0.197-0.532) | **<0.001** |
| 1p/19q codeletion | 527 |  |  |  |  |  |
| codel | 170 | Reference |  |  |  |  |
| non-codel | 357 | 2.493 (1.590-3.910) | **<0.001** |  | 1.925 (1.122-3.302) | **0.017** |

**Univariate analysis and multivariate analysis for *TP53* in OS of LGG**

| Characteristics | Total(N) | Univariate analysis | |  | Multivariate analysis | |
| --- | --- | --- | --- | --- | --- | --- |
|  |  | Hazard ratio (95% CI) | P value |  | Hazard ratio (95% CI) | P value |
| WHO grade | 466 |  |  |  |  |  |
| G2 | 223 | Reference |  |  |  |  |
| G3 | 243 | 3.059 (2.046-4.573) | **<0.001** |  | 1.955 (1.256-3.042) | **0.003** |
| TP53 | 527 |  |  |  |  |  |
| Low | 264 | Reference |  |  |  |  |
| High | 263 | 1.689 (1.189-2.400) | **0.003** |  | 1.589 (1.053-2.397) | **0.027** |
| Age | 527 |  |  |  |  |  |
| <=40 | 264 | Reference |  |  |  |  |
| >40 | 263 | 2.889 (2.009-4.155) | **<0.001** |  | 3.202 (2.088-4.911) | **<0.001** |
| Gender | 527 |  |  |  |  |  |
| Female | 238 | Reference |  |  |  |  |
| Male | 289 | 1.124 (0.800-1.580) | 0.499 |  |  |  |
| IDH status | 524 |  |  |  |  |  |
| WT | 97 | Reference |  |  |  |  |
| Mut | 427 | 0.186 (0.130-0.265) | **<0.001** |  | 0.348 (0.220-0.552) | **<0.001** |
| 1p/19q codeletion | 527 |  |  |  |  |  |
| codel | 170 | Reference |  |  |  |  |
| non-codel | 357 | 2.493 (1.590-3.910) | **<0.001** |  | 2.016 (1.201-3.385) | **0.008** |

**Univariate analysis and multivariate analysis for *CASP9* in OS of ACC**

| Characteristics | Total(N) | Univariate analysis | |  | Multivariate analysis | |
| --- | --- | --- | --- | --- | --- | --- |
|  |  | Hazard ratio (95% CI) | P value |  | Hazard ratio (95% CI) | P value |
| T stage | 77 |  |  |  |  |  |
| T1&T2 | 51 | Reference |  |  |  |  |
| T3&T4 | 26 | 10.286 (3.976-26.608) | **<0.001** |  | 7.936 (0.937-67.214) | 0.057 |
| N stage | 77 |  |  |  |  |  |
| N0 | 68 | Reference |  |  |  |  |
| N1 | 9 | 2.038 (0.769-5.400) | 0.152 |  |  |  |
| M stage | 77 |  |  |  |  |  |
| M0 | 62 | Reference |  |  |  |  |
| M1 | 15 | 6.150 (2.710-13.959) | **<0.001** |  | 2.519 (0.936-6.775) | 0.067 |
| CASP9 | 79 |  |  |  |  |  |
| Low | 39 | Reference |  |  |  |  |
| High | 40 | 4.849 (1.959-11.998) | **<0.001** |  | 5.087 (1.846-14.020) | **0.002** |
| Pathologic stage | 77 |  |  |  |  |  |
| Stage I&Stage II | 46 | Reference |  |  |  |  |
| Stage III&Stage IV | 31 | 6.476 (2.706-15.498) | **<0.001** |  | 0.616 (0.076-5.001) | 0.651 |
| Age | 79 |  |  |  |  |  |
| <=50 | 41 | Reference |  |  |  |  |
| >50 | 38 | 1.799 (0.846-3.824) | 0.127 |  |  |  |
| Gender | 79 |  |  |  |  |  |
| Female | 48 | Reference |  |  |  |  |
| Male | 31 | 1.001 (0.469-2.137) | 0.999 |  |  |  |
